# Proteome analysis reveals a role of rainbow trout lymphoid organs during *Yersinia ruckeri* infection process

**DOI:** 10.1038/s41598-018-31982-6

**Published:** 2018-09-18

**Authors:** Gokhlesh Kumar, Karin Hummel, Katharina Noebauer, Timothy J. Welch, Ebrahim Razzazi-Fazeli, Mansour El-Matbouli

**Affiliations:** 1 Clinical Division of Fish Medicine, University of Veterinary Medicine, Vienna, Austria; 2 VetCore Facility for Research/Proteomics Unit, University of Veterinary Medicine, Vienna, Austria; 3 National Center for Cool and Cold Water Aquaculture, Kearneysville, USA

## Abstract

*Yersinia ruckeri* is the causative agent of enteric redmouth disease in salmonids. Head kidney and spleen are major lymphoid organs of the teleost fish where antigen presentation and immune defense against microbes take place. We investigated proteome alteration in head kidney and spleen of the rainbow trout following *Y*. *ruckeri* strains infection. Organs were analyzed after 3, 9 and 28 days post exposure with a shotgun proteomic approach. GO annotation and protein-protein interaction were predicted using bioinformatic tools. Thirty four proteins from head kidney and 85 proteins from spleen were found to be differentially expressed in rainbow trout during the *Y*. *ruckeri* infection process. These included lysosomal, antioxidant, metalloproteinase, cytoskeleton, tetraspanin, cathepsin B and c-type lectin receptor proteins. The findings of this study regarding the immune response at the protein level offer new insight into the systemic response to *Y*. *ruckeri* infection in rainbow trout. This proteomic data facilitate a better understanding of host-pathogen interactions and response of fish against *Y*. *ruckeri* biotype 1 and 2 strains. Protein-protein interaction analysis predicts carbon metabolism, ribosome and phagosome pathways in spleen of infected fish, which might be useful in understanding biological processes and further studies in the direction of pathways.

## Introduction

Enteric redmouth disease (ERM) causes significant economic losses in salmonids worldwide. The disease is caused by *Yersinia ruckeri*, a Gram negative rod-shaped enterobacterium. Rainbow trout (*Oncorhynchus mykiss*) are especially susceptible to ERM, although infections have been reported in other fish species^1,2^. The signs of the disease include exophthalmia and darkening of the skin, and subcutaneous hemorrhages in and around the mouth and throat. The spleen is often enlarged and can be almost black in color^1^. Multifocal necrosis can be seen in the spleen, and degenerated renal tubules, glomerular nephritis and a marked increase in melano-macrophages can be observed in the kidney of fish infected with *Y*. *ruckeri*^1,2^.

*Y*. *ruckeri* strains are classified into two biotypes: biotype 1 strains are positive for motility and lipase secretion, whereas biotype 2 strains are non-motile and negative for lipase secretion. Biotype 2 strains have been responsible for outbreaks in naïve and vaccinated rainbow trout that have been vaccinated against biotype 1 strains^3^. However, bivalent or biotype 2 vaccines provide good protection in rainbow trout against the biotype 2 strains^4^.

The head kidney and spleen are major lymphoid organs in the teleost fish where antigen presentation and immune defense against microbes take place^5^. These lymphoid organs have been shown to exert an active role in immune response of rainbow trout against *Y*. *ruckeri*. Regulation of non-specific and specific immune genes such as pro-inflammatory cytokines, chemokines and cell receptors have largely been studied in rainbow trout after experimental challenge with *Y*. *ruckeri*^6–9^. Recently, RNA-seq analysis has been used to detect changes in gene expression following *Y*. *ruckeri* strain H01 challenge of Amur sturgeon (*Acipenser schrenckii*)^10^. Although these research studies are valuable, they are based on mRNA gene expression levels, which may not accurately reflect protein expression. In addition, post-translational modifications cannot be determined by mRNA analysis^11^.

Proteomics can provide significant information on biochemical changes in organisms that is absent in the transcriptome^12^. The mechanisms of bacterial pathogenesis and the biochemical changes in cellular protein expression in infected host still require examination. More recently, we identified differentially expressed proteins and global proteomic profiles of *Y*. *ruckeri* strains in order to elucidate proteomic biology and changes between strains^13,14^. There is an urgent need to understand the protein changes in the host lymphoid organs in response to *Y*. *ruckeri* infection.

The aim of the present study was to identify and quantify rainbow trout lymphoid organ proteomic expression profiles in response to infection with biotype 1 and biotype 2 *Y*. *ruckeri* strains by SWATH-MS (Sequential Windowed Acquisition of All Theoretical Mass Spectra), a label-free quantitative proteomic approach.

## Results

### Fish mortality and *Y. ruckeri* infection

**Figure 1:**
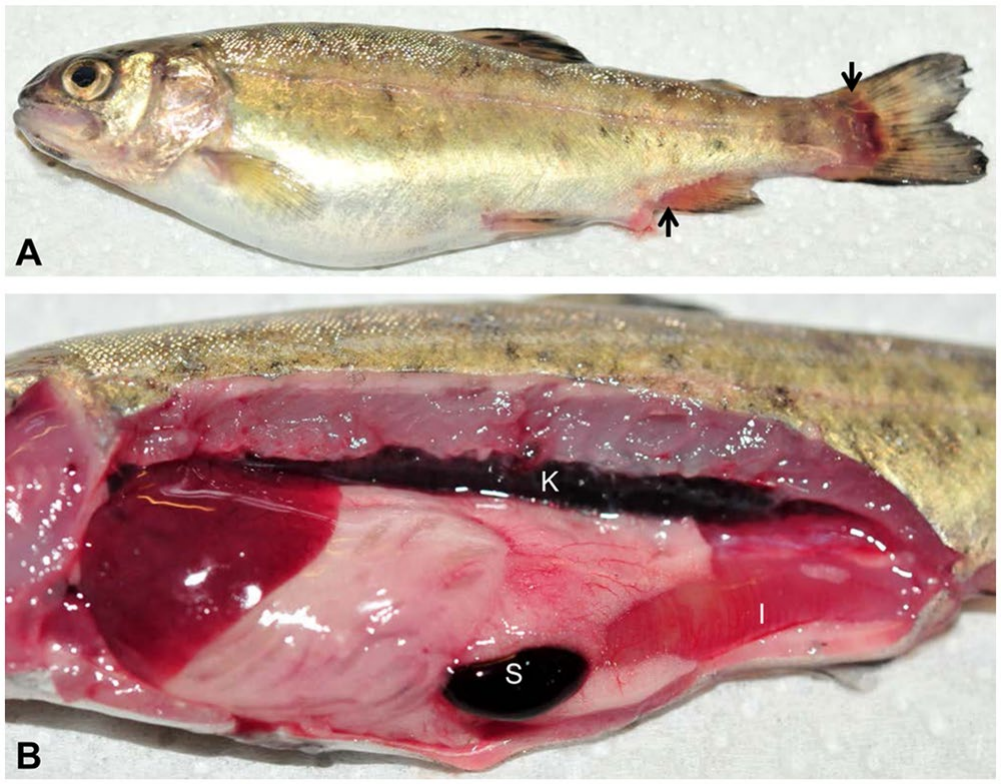
Rainbow trout showing clinical signs of enteric redmouth disease. (**A**) Hemorrhages in the caudal and anal fins (arrows). (**B**) Enlarged and black spleen and reddened intestine. Note: S: spleen, K: kidney and I: intestine.

*Y*. *ruckeri* was re-isolated from the head kidney of all dead fish during the experimental periods. Dead fish exhibited gross disease signs typical of ERM infection (Fig. 1). During the 28 day period following the infection 30% of the infected fish died and most of the mortality was recorded between 8–10 days post exposure (dpe). We observed enlarged spleen and reddened intestine at 9 dpe. No mortality occurred in the control group and no bacteria were isolated from the head kidney of the control group when sampled at 3, 9 and 28 dpe.

### Differentially expressed proteins

**Figure 2:**
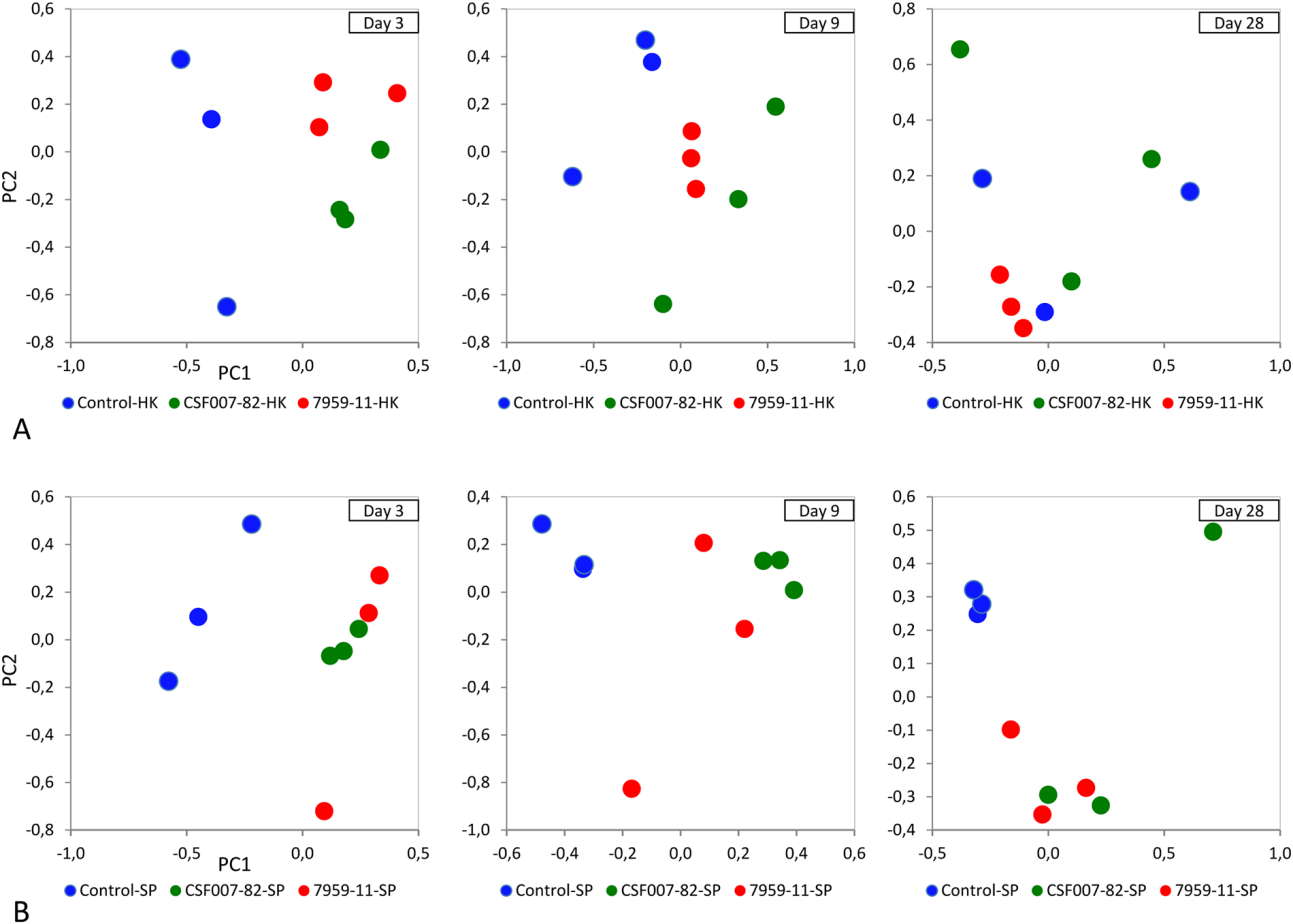
Principle component analysis of head kidney and spleen samples. Two dimensional map of the principal component PC1–PC2 showed all three replicates of three groups (Control, CSF007-82-infected and 7959-11-infected) in a plot at 3, 9 and 28 days post exposure. (**A)** Head kidney proteins; B: spleen proteins.

Principal component analysis (PCA) plots provide an overview of the complex proteomics data of head kidney and spleen samples at 3, 9 and 28 dpe, showing a clear separation between control and infected organ samples at each time point with exception of head kidney at 28 dpe (Fig. 2). This data suggests that statistically significant differential expression is responsible for the clustering of the samples.

Statistical evaluation revealed 34 differentially regulated proteins (32 up and 2 down-regulated proteins) in the head kidney (Supplementary Table S1) and 85 differentially regulated proteins (68 up and 17 down-regulated proteins) in the spleen (Supplementary Table S2) of rainbow trout in response to *Y*. *ruckeri* infection at different time points. Down-regulated proteins were mainly found in the infected spleen at 3 dpe (Supplementary Table S2). Most of them were uncharacterized proteins. These were homologous to apolipoprotein B-100 isoform X3 (90.2%), collagen alpha-3(VI) chain-like isoform X1 (72.8%), lumican (98.8%), nucleoprotein TPR-like isoform X2 (73.5%), non-muscle caldesmon-like isoform X7 (97.9%), ependymin (97.2%) and laminin subunit beta-2-like (98.8%).

**Figure 3:**
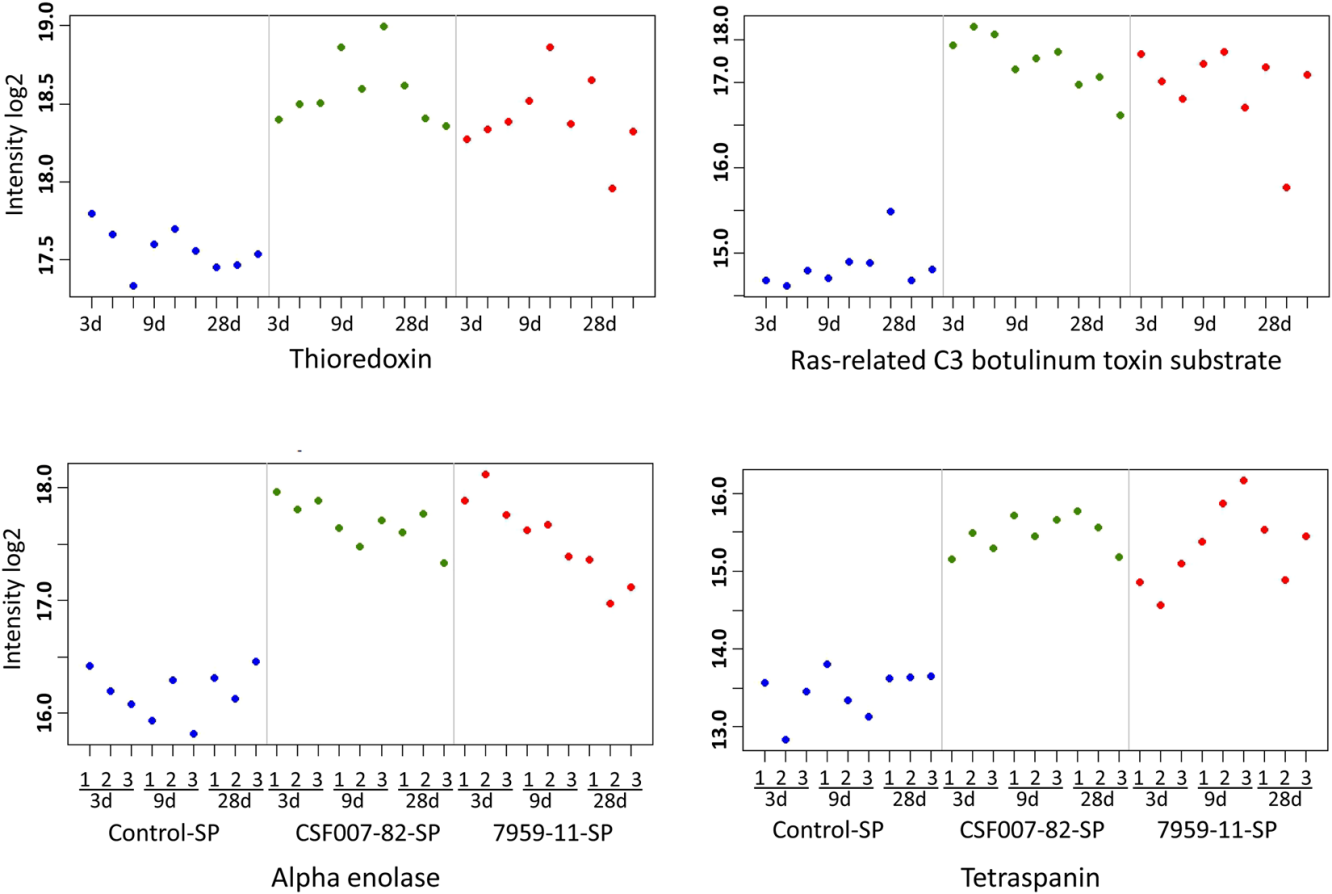
Expression plot of top candidate spleen proteins. Plot showed protein expression (n = 3 pools of 9 individual biological replicates per time point) of thioredoxin, ras-related C3 botulinum toxin substrate 2, alpha enolase and tetraspanin at 3, 9 and 28 days post exposure.

The top differentially expressed proteins of head kidney and spleen are presented in Tables 1 and 2, respectively. The expression of top candidate spleen proteins is presented in Fig. 3. It can be seen clearly that the control samples of spleen at all time points show lower amounts of thioredoxin, ras-related C3 botulinum toxin substrate 2, alpha enolase, and tetraspanin compared to all infected samples. Biotypes 1 and 2 strains induced minor proteomic changes in the head kidney and spleen of infected rainbow trout (Supplementary Tables S1 and S2).

**Table 1:** List of top up- and down-regulated head kidney proteins of rainbow trout in response to *Yersinia ruckeri* strains. Fold change (infected vs control) was statistically analysed in *Y*. *ruckeri* CSF007-82 (biotype 1) and 7959-11 (biotype 2) infected and control rainbow trout samples (n = 27).

Accession UniProt	Protein	Number of quantified Peptides	Function	Head kidney control in response to strain	3 dpe	9 dpe	28 dpe
LYSC2_ONCMY	Lysozyme C II	6	Lysozyme activity	CSF007-82	2.2	4.8*	2.2
				7959-11	2.0	4.8*	1.2
Q68S98_SALSA	C type lectin receptor A	2	Carbohydrate binding	CSF007-82	2.6*	4.8*	1.4
				7959-11	2.3*	3.6*	1.2
C0KIP4_ONCMY	C type lectin receptor B	4	Carbohydrate binding	CSF007-82	2.1*	4.3*	2.1
				7959-11	2.0*	2.8*	1.5
Q5DVP5_ONCMY	Interleukin-16 (Fragment)	2	Cytokine activity	CSF007-82	2.1*	2.1	2.2
				7959-11	2.3*	2.3	1.6
Q9PT14_ONCMY	Precerebellin-like protein	5	Response to lipopolysaccharide	CSF007-82	2.9	10.8*	2.9
				7959-11	2.5	6.4*	1.9
Q9DFJ1_ONCMY	Chemotaxin (Fragment)	5	Neutrophil chemotactic activity	CSF007-82	4.1*	2.9	1.1
				7959-11	2.7*	2.1	−1.3
B5XDZ7_SALSA	Catechol-O-methyltransferase domain-containing protein 1	5	O-methyltransferase activity	CSF007-82	2.4*	6.6*	2.3
				7959-11	2.2*	3.3*	1.4
B5X215_SALSA	Ribonucleoside-diphosphate reductase subunit M2	4	Ribonucleoside-diphosphate reductase activity	CSF007-82	2.3*	2.2	1.1
				7959-11	2.2*	1.5	−1.3
I3WWD7_ONCMY	Plasminogen activator inhibitor 1	2	Serine protease inhibitor	CSF007-82	3.2*	1.4	1.2
				7959-11	2.9*	1.3	−1.1
C1BHS7_ONCMY	Protein S100	2	Calcium ion binding	CSF007-82	−1.4	−2.2*	−1.1
				7959-11	−1.4	−1.6	1.1
W8W0Y8_ONCMY	Glutathione peroxidase	6	Anti-oxidant activity	CSF007-82	−1.9	−2.6*	−1.3
				7959-11	−2.5	−1.4	−1.1

*Denotes statistically significant difference according to both ANOVA and post hoc Tukey’s HSD with FDR-adjusted *p*-value < 0.05 and fold change <−2 or >+ 2. (Full table is presented in Supplementary Table S1).

**Table 2:** List of top up- and down-regulated spleen proteins of rainbow trout in response to *Yersinia ruckeri* strains.

Accession UniProt	Protein	Number of quantified Peptides	Function	Spleen control in response to strain	3 dpe	9 dpe	28 dpe
LYSC2_ONCMY	Lysozyme C II	6	Lysozyme activity	CSF007-82	4.6*	11.8	3.7
				7959-11	3.0*	6.6	3.5
Q60FB6_ONCMY	NADPH oxidase cytosolic protein p40phox	6	Phagocytosis	CSF007-82	3.0*	2.6	1.5
				7959-11	3.0*	2.2	1.3
Q60FB5_ONCMY	NADPH oxidase cytosolic protein p67phox	4	Phagocytosis	CSF007-82	2.7*	2.2	−1.3
				7959-11	3.1*	2.1	−1.4
C1BHL9_ONCMY	Ras-related C3 botulinum toxin substrate 2	2	Phagocytosis /GTPase activity	CSF007-82	7.3*	5.4*	3.6
				7959-11	5.2*	4.9*	3.4
C1BH85_ONCMY	Thioredoxin	5	Antioxidant defence	CSF007-82	3.2*	4.8	3.6
				7959-11	2.8*	2.6	3.1
W8W0Y8_ONCMY	Glutathione peroxidase	5	Anti-oxidant activity	CSF007-82	1.9	1.3	1.6
				7959-11	2.2*	1.9	1.4
Q92004_ONCMY	Beta-2-microglobulin	4	Glycoprotein binding	CSF007-82	2.2	4.1*	2.0
				7959-11	1.7	3.0*	2.1
Q9DFJ1_ONCMY	Chemotaxin (Fragment)	5	Neutrophil chemotactic activity	CSF007-82	4.9*	3.6	1.5
				7959-11	4.2*	3.0	1.3
A0A060X145_ONCMY	Tetraspanin	2	Cell surface receptor signaling pathway	CSF007-82	4.0*	4.5*	3.7*
				7959-11	2.9*	5.2*	3.2*
B5X4P4_SALSA	Cathepsin B	2	Cysteine-type endopeptidase activity	CSF007-82	2.3	3.8*	2.6*
				7959-11	1.5	2.9*	2.6*
B9ENC0_SALSA	Cellular nucleic acid-binding protein	5	Nucleic acid binding	CSF007-82	5.4*	7.2*	5.7*
				7959-11	4.5*	7.1*	4.3*
C1BEZ5_ONCMY	C6orf115	2	Protein folding	CSF007-82	3.0*	4.0*	2.8*
				7959-11	2.5*	2.5*	2.2*
C1BH21_ONCMY	Dynein light chain 1, cytoplasmic	3	Microtubule-based process	CSF007-82	2.0	2.9*	2.4*
				7959-11	1.7	2.9*	2.1*
B5X1B5_SALSA	Alpha-enolase	2	Glycolytic process	CSF007-82	3.1*	3.0*	2.4*
				7959-11	3.2*	2.9*	1.8
C1BHS7_ONCMY	Protein S100	2	Calcium ion binding	CSF007-82	−1.3	−1.5	−1.1
				7959-11	−2.4*	−1.4	1.2

Details as in Table 1. (Full table is presented in Supplementary Table S2).

### GO annotation

The identified up and down-regulated proteins were associated with several biological processes: immune system, cellular, metabolic, developmental, multicellular, adhesion, regulation, interaction, localization, and response to stimulus. These proteins were associated with extracellular, plasma membrane, cytoplasm, cytoskeleton, nucleus, ribosome and macromolecular complex. These proteins were involved in binding, catalytic, receptor, antioxidant and structural molecular activities. Additionally, these proteins were involved in several pathways: T cell activation, apoptosis signaling, oxidative stress response (phagosome), blood coagulation and pentose phosphate pathways.

### Protein-protein interaction

**Figure 4:**
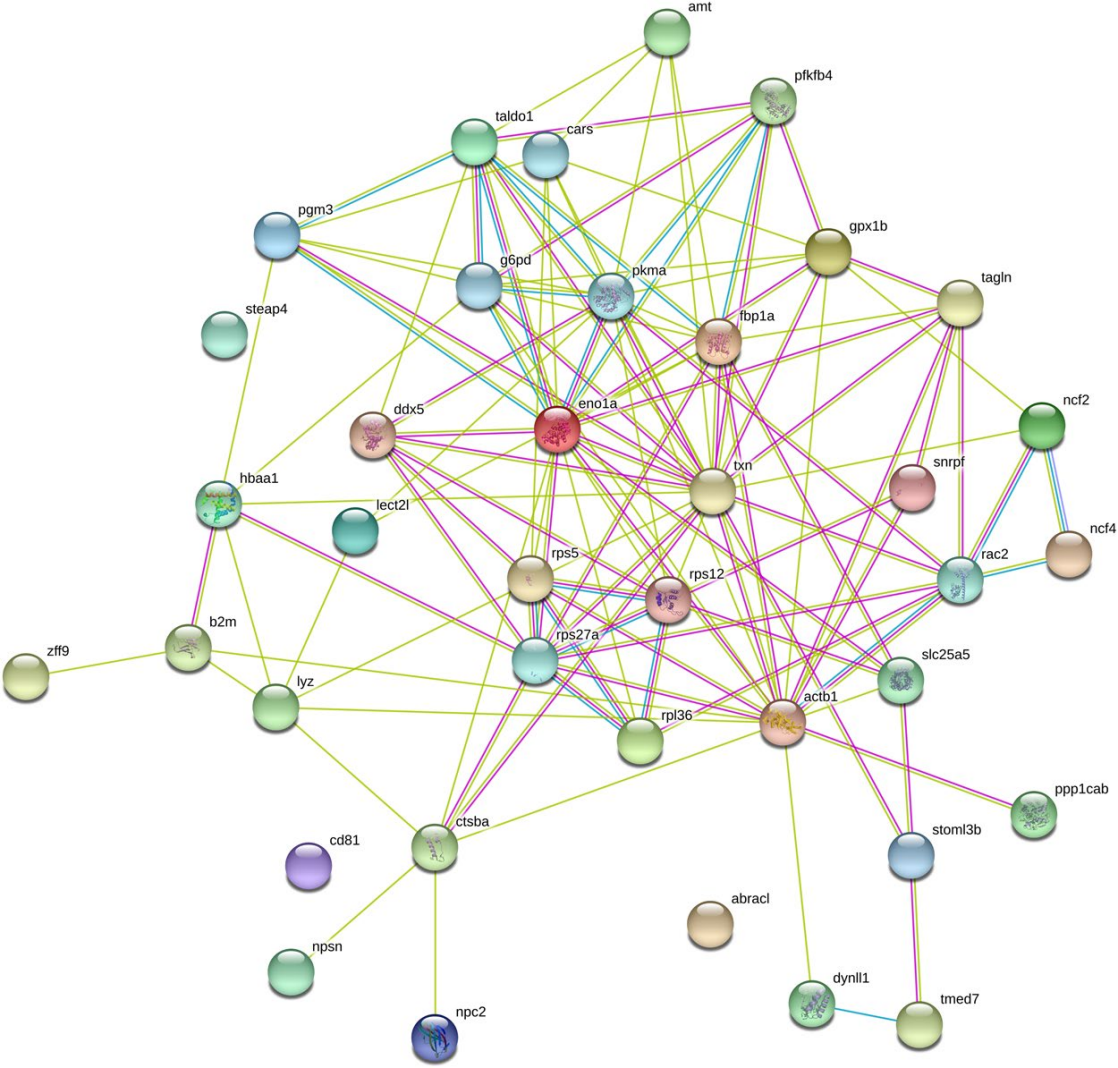
The protein-protein interaction network of 38 up-regulated spleen proteins excluding uncharacterized proteins. In this network, nodes are proteins, lines represent the predicted functional associations, and the number of lines represents the strength of predicted functional interactions between proteins. The yellow lines represent textmining evidence; the purple lines represent experimental evidence and the light blue lines represent database evidence.

As shown in Fig. 4, out of 38, 35 proteins up-regulated in infected spleen were predicted to be involved in protein-protein interactions. Thirty one proteins including alpha-enolase, transaldolase, transgelin, ribosomal protein S5, ribosomal protein S12, beta-actin, hemoglobin, thioredoxin, cathepsin B and glutathione peroxidase showed high connectivity in the predicted protein-protein interaction analysis. The data showed that alpha-enolase and thioredoxin were the central node of protein–protein interaction analysis. Three clusters of protein association network were obtained in the spleen by using STRING analysis. These clusters were involved in the carbon metabolism, ribosome and phagosome KEGG pathways. The head kidney proteins did not show very well connection to each other in protein-protein interaction analysis (data not shown).

### Transcriptional regulation of select genes

**Figure 5:**
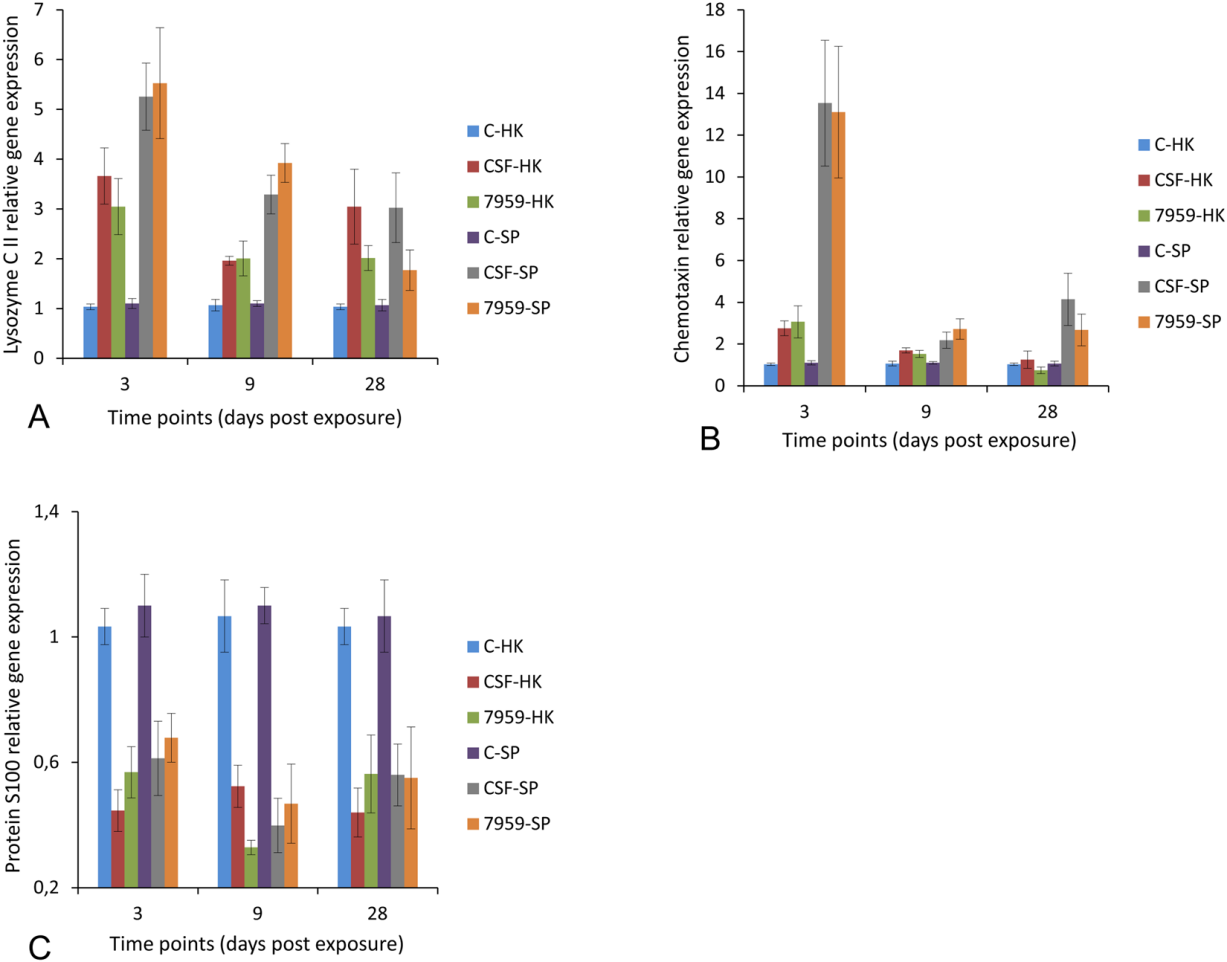
Relative expression levels of lysozyme C II, chemotaxin and protein S100 in rainbow trout. Quantitative real-time PCR showing mean relative expression profiles of each selected gene in head kidney (HK) (n = 27) and spleen (SP) (n = 27) samples in response to *Y*. *ruckeri* CSF007-82 (biotype 1) and 7959-11 (biotype 2) at different time points. Relative gene expression changes of each gene were determined in infected and control samples for each group at each time point by calculating the mean normalized expression values of each organ at each time point. Error bars indicate standard deviation. (**A)** Lysozyme C II, (**B**) Chemotaxin and (**C**): Protein S100. (**C**) Control fish (mock infected), CSF: *Y*. *ruckeri* CSF007-82-infected fish and 7959: *Y*. *ruckeri* 7959-11-infected fish.

**Figure 6:**
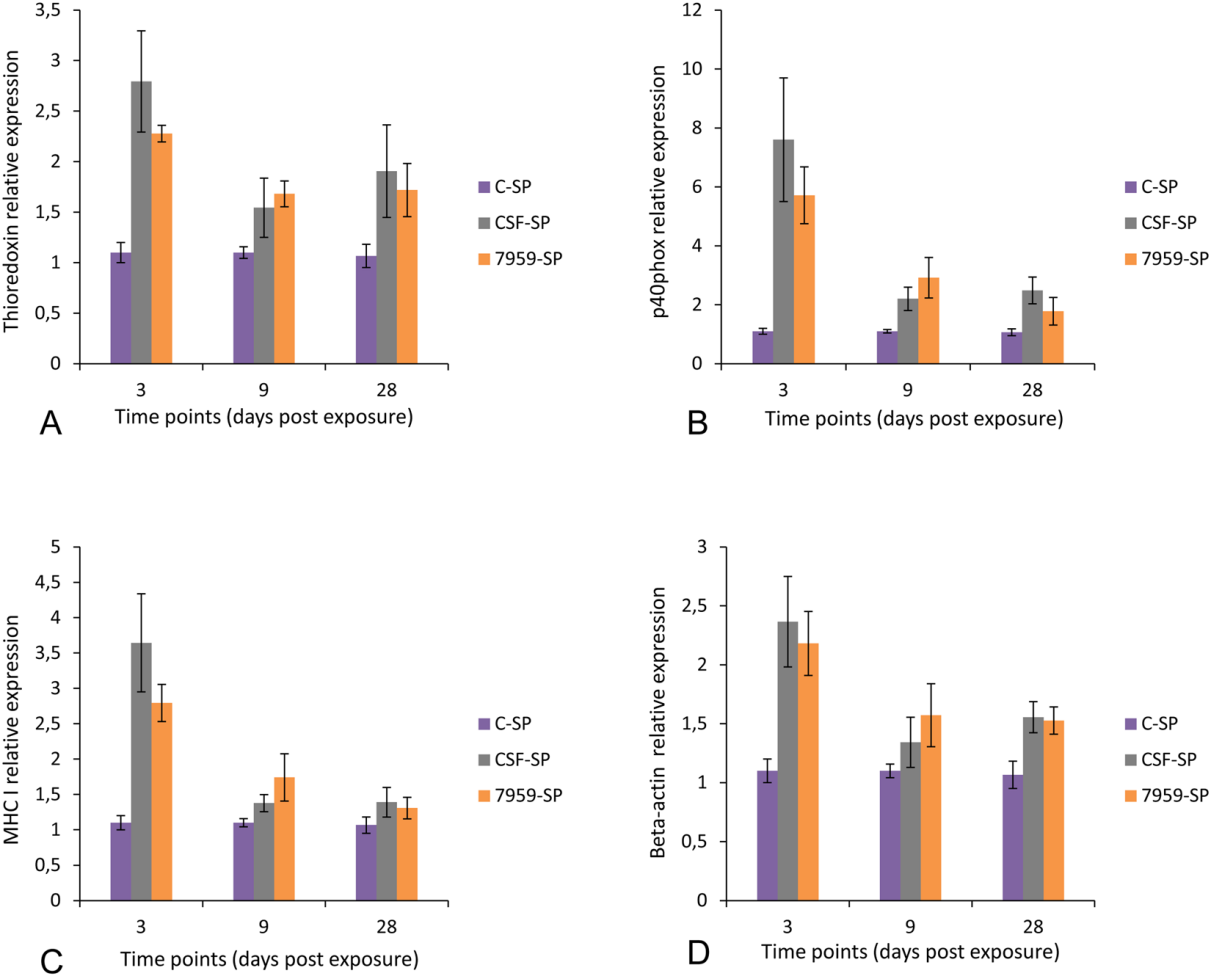
Relative expression levels of thioredoxin, p40phox, MHC I and beta-actin in rainbow trout. Quantitative real-time PCR showing mean relative expression profiles of each selected gene in spleen (SP) (n = 27) samples in response to *Y*. *ruckeri* CSF007-82 (biotype 1) and 7959-11 (biotype 2) at different time points. Relative gene expression changes of each gene were determined in infected and control samples for each group at each time point by calculating the mean normalized expression values of each organ at each time point. Error bars indicate standard deviation. (**A**) Thioredoxin, (**B**) p40phox, (**C**) MHC I and (**D**) beta-actin. (**C**) Control fish (mock infected), CSF: *Y*. *ruckeri* CSF007-82-infected fish and 7959: *Y*. *ruckeri* 7959-11-infected fish.

Differentially expressed proteins identified in the MS analysis were examined by quantitative real time PCR to determine the role of transcriptional regulation in the observed differential protein expression patterns (Figs 5 and 6). All selected genes coding differentially expressed proteins were up-regulated (*p* < 0.0001) in the infected samples, relative to the control samples. The candidate proteins were selected on the basis of their differential expression as representative of lysozyme activity, neutrophil chemotactic activity, antioxidant defence, phagocytosis, antigen presentation, actin filament binding and calcium ion binding. The results demonstrated that the regulation of these genes was consistent with corresponding quantitative proteomic data. However, the level of mRNA expression slightly varied in parallel to the corresponding protein expression perhaps suggesting additional post-transcriptional regulation (Figs 5 and 6).

## Discussion

Bacterial infection and the resulting host response involve a complex interaction network in which bacteria subvert host cell processes to increase the efficiency of infection. Similarly the host responds to infection in an attempt to limit pathogen proliferation and spread^15^. Head kidney and spleen are known to be important lymphoid organs for both innate and adaptive immunity and host-pathogen interaction^5^. Herein we have used recent innovation of SWATH-MS technology, for the first time, to elucidate quantitative proteomic profiles from head kidney and spleen obtained from rainbow trout infected with *Y*. *ruckeri* biotypes 1 and 2 strains. We present here an accurate measurement and comprehensive interpretation of protein expression in the head kidney and spleen of infected rainbow trout at different time points.

The interaction of a bacterial pathogen with its host leads to a variety of physiological responses aimed at an adjusting to the new condition^15^. In the present study, we found up-regulation of some metabolic proteins [alpha-enolase (2.4 to 3.2 fold), fructose-1,6-bisphosphatase 1 (2.5 to 4.0 fold) and glucose-6-phosphate 1-dehydrogenase (2.3 to 3.0 fold)] and amino acid proteins [cellular nucleic acid-binding protein (4.3 to 7.2 fold), small nuclear ribonucleoprotein F (2.1 to 2.8 fold), ribosomal protein S5 (2.7 to 3.7 fold) and C6orf115 (2.2 to 4.0 fold)] in spleen of rainbow trout in response to *Y*. *ruckeri* infection at different time points (Table 2). It is unclear what role these metabolic proteins may have in immunity of fish against infection. Interestingly, up-regulation of alpha-enolase (1.8 to 17.6 fold) and fructose-bisphosphate aldolase C (5.3 fold) are observed by using 2D-DIGE analysis in the fins of viral haemorrhagic septicemia rhabdovirus infected-zebrafish^16^ and in the spleen of *Edwardsiella tarda* infected-Japanese flounder (*Paralichthys olivaceus*)^17^. These observations suggest that an enhanced metabolic activity might be required for efficient antigen processing and presentation by fish to stimulate a robust immune response during *Y*. *ruckeri* infection. However, further characterization of some metabolic proteins will be needed to fully understand the immune response of fish during bacterial infection.

Lysozymes are important proteins of the innate immune system and first line of defense against pathogens^18^. We consistently found up-regulation of lysozyme C II in the head kidney (Table 1) and spleen (Table 2) of rainbow trout in response to *Y*. *ruckeri* strains at 3–9 dpe. The up-regulation of lysozyme C II was much stronger in spleen (3.0 to 11.8 fold) than that of head kidney (2.2 to 4.8 fold) of infected rainbow trout. The expression of c-type lysozyme was up-regulated (7.2 fold) in the liver of taimen (*Hucho taimen*, Pallas) 24 hours post *Y*. *ruckeri* infection^18^. Additionally, we found significant up-regulation of cathepsin B (2.3 to 3.8 fold) in the spleen of infected rainbow trout at 9 and 28 dpe (Table 2). Cathepsin B is a major lysosomal cysteine protease and plays an important role in intracellular proteolysis. It also up-regulates the activity of other proteases including matrix metalloproteinase^19^, where we found up-regulation of metalloendopeptidase in infected rainbow trout with cathepsin B, thereby suggesting that cathepsin B may play a multifunctional role in immune response in fish during bacterial infection. Finally, these results suggest that lysosomal proteins undergo proteolytic processing steps within the endosomal compartment, which provide antibacterial defense mechanism in the fish during bacterial infection.

Reactive oxygen species lead to oxidative stress in cells and contribute to the elimination of microbial infections, including the immune response^20^. We found significant up-regulation of antioxidant systems such as glutathione peroxidase (2.2 fold), NADPH oxidase cytosolic proteins [p40phox (2.2 to 3.0 fold) and p67phox (2.1 to 3.1 fold)], ras-related C3 botulinum toxin substrate 2 (3.4 to 7.3 fold) in infected spleen at 3 dpe but not in infected head kidney. Glutathione peroxidase reduces hydrogen peroxide in the cell during inflammatory response to protect the organism from oxidative stress^20,21^. NADPH oxidase is a membrane-bound enzyme complex and generates superoxide anions. It is made up of six subunits: Rac1 or Rac2 (Rac stands for Rho-related C3 botulinum toxin substrate), gp91-PHOX, p22phox, p40phox, p47phox and p67phox^21^. These cytosolic proteins (p40phox and ras-related C3 botulinum toxin substrate) were up-regulated in rainbow trout in response to infectious hematopoietic necrosis virus DNA vaccine^22^ and *Escherichia coli* lipopolysaccharide^23^. This suggests an involvement of the phagosome pathway in the spleen of infected rainbow trout. Additionally, we found up-regulation of thioredoxin (2.6 to 4.8 fold) in the spleen of infected rainbow trout. Thioredoxin is the essential component of the thioredoxin system and is one of the central reducing antioxidant systems in all organisms^24^. The expression of thioredoxin genes (*Trx1* and *Trxr3a*) was observed in lymphoid organs of *Y*. *ruckeri*-infected rainbow trout at 24–72 hours post infection^25^. These results suggest that the respiratory burst of infected fish is induced in order to eliminate the bacteria, and perhaps play an important role in maintaining cellular redox homeostasis and induce the innate immune system in rainbow trout during *Y*. *ruckeri* infection.

*STEAP4*, a gene encoding a plasma membrane metallo-reductase, is involved in the transport of iron and in the control of inflammatory cytokines^26^. Up-regulation of *STEAP4* (2.8 to 5.3 fold) was observed in spleen of Atlantic cod (*Gadus morhua*) in response to formalin-killed *Aeromonas salmonicida*^27^. We also found up-regulation of metalloreductase STEAP4 (2.2 to 3.7 fold) in spleen of rainbow trout in response to *Y*. *ruckeri* at 3 and 9 dpe (Supplementary Table 2). Additionally, metalloendopeptidase was up-regulated (3.1 to 4.7 fold) in infected spleen of rainbow trout at 3 and 28 dpe (Supplementary Table 2). Matrix metalloproteinases are inflammation-responsive proteins and belong to a family of zinc-dependent endopeptidases^28^. These are involved in degradation of extracellular matrix, tissue repair, cellular signaling, inflammation and immune response in different fish species^28^. Matrix metalloproteinase-9 is suggested to be involved in protecting zebrafish against *Listeria monocytogene*s infection by engaging in macrophage migration to the site of infection to exert their phagocytic activity^29^. This is in agreement with the finding of Raida and Buchmann^6^, who found increased levels of interleukin-8 (IL-8) mRNA expression in the spleen of *Y*. *ruckeri***-**infected rainbow trout. IL-8 is a key molecule for the regulation of inflammation and immune responses in fish. Up-regulation of metalloreductase and metalloproteinase proteins suggests a role for these proteins in the establishment of inflammatory and immune response in rainbow trout during *Y*. *ruckeri* infection.

S100 is a calcium ion binding protein that is involved in the control of cell growth and proliferation, cell cycle progression and modulation of specific signal transduction pathways^30^. Protein S100 was significantly down-regulated (−2.2 to −3.2 fold) in both head kidney and spleen of *Y*. *ruckeri-*infected rainbow trout at 3 dpe. Down-regulation of the S100-A1 protein (−2.2 fold) was also observed in head kidney of *Enteromyxum leei* infected-gilthead sea bream (*Sparus aurata*) when assessed by cDNA microarray^31^. Based on our proteome results, we suggest that *Y*. *ruckeri* may capture and reduce the activities of some ion binding proteins in the spleen of infected fish.

The cytoskeleton is a complex network of interlinking filaments and tubules whose functions include maintenance of cellular shape, cellular migration, division, intracellular transport, signaling and host defense mechanisms^32^. Up-regulation of cytoskeleton-related proteins such as beta-actin (2.1 fold), transgelin (2.4 fold) and dynein light chain 1 (2.1 to 2.9 fold) was observed in spleen of infected rainbow trout at different time points. Actin has been reported to interact with lipoteichoic acid of Gram-positive bacteria and interfere with bacterial entry into host cells^33,34^. The up-regulation (3.6 fold) of beta-actin was observed in the spleen of Japanese flounder in response to *E*. *tarda* at 12 hours post infection^17^, which is consistent with our results showing a 2.1 fold increase in protein expression of beta-actin in the spleen of rainbow trout in response to *Y*. *ruckeri* at 3 dpe. Transgelin is a member of the calponin-family of actin-binding proteins and has been named SM22^35^. Transgelin is up-regulated in head kidney (3.1 fold) of common carp infected with cyprinid herpesvirus 3 at 3 dpi^36^. Expression of cytoskeleton-related proteins observed in our study suggests that these proteins might play a role in the host defense. Further research is needed to confirm the role of cytoskeleton-related proteins in the immune response of fish against bacterial infection.

We found for the first time, that tetraspanin was consistently up-regulated (2.9 to 5.2 fold) in spleen of rainbow trout in response to *Y*. *ruckeri* strains at 3–28 dpe. Tetraspanin family members in rainbow trout are homologous to mammalian CD9 and CD63. Tetraspanins are a family of membrane-organizing proteins that mediate diverse functions including immune functions^37^. This suggests that tetraspanin may act as immune-related proteins against pathogen infection. Additionally, we found non-significant (*p* = 0.06) up-regulation of major histocompatibility complex (MHC) class I (2.2 to 2.9 fold) at 3 dpe and significant (*p* < 0.05) up-regulation of beta 2-microglobulin (3 to 4.1 fold) at 9 dpe in spleen during *Y*. *ruckeri* infection. However, we confirmed significant (*p* < 0.001) up-regulation of MHC class I (>2.7 fold) in infected spleen at the transcriptional level by RT-qPCR (Fig. 6C). MHC class I molecules represent a peptide fragment of a specific antigen to cytotoxic T cells and consist of two polypeptide chains, α and β2-microglobulin. Li *et al*.^10^ found up-regulation of MHC class I (3.5 fold) in *Y*. *ruckeri*-infected spleen of Amur sturgeon at 24 hours post challenge. These authors also identified several relevant components of the T cell receptor (TCR) signaling pathway in spleen by analyzing the global transcriptome profiles of Amur sturgeon. These findings suggest that the TCR signaling pathway may be involved in the regulation of immune response of rainbow trout against *Y*. *ruckeri*.

We found up-regulation of interleukin-16 (2.1 to 2.3 fold), interferon-induced guanylate-binding protein 1 (2.4 fold) and chemotaxin (2.1 to 4.9 fold) in head kidney of infected rainbow trout. Interleukin-16 is a pro-inflammatory cytokine that functions in chemotaxis for CD4^+^ T lymphocytes^38^. Interferon-induced guanylate-binding protein 1 belongs to the dynamin superfamily of large GTPases and has been shown to mediate the antibacterial and antiviral activities of gamma interferon^39^. Interferon-induced transmembrane protein 1-like is expressed in the spleen of *Y*. *ruckeri*-infected Amur sturgeon at 24 hours post challenge^10^. Chemotaxin is a chemotactic protein that releases chemotactic factors from inflammatory sites and plays an important role in activating neutrophil functions^40^. Wei *et al*.^41^ found expression of chemotaxin-2 (*EcLECT2*) (50 to 320 fold) in the liver of marine fish grouper (*Epinephelus coioides*) at 24 hours after infection with different microbes. We also identified uncharacterized protein (A0A060YAR2_ONCMY) that was up-regulated in head kidney (3.9 to 4.8 fold) at 9 dpe and in the spleen at 3 dpe (5.3 to 7.7 fold) and at 9 dpe (5.3 to 7.7 fold). This uncharacterized protein has 88.7% similarity to interferon-induced protein 44-like isoform X2 (A0A1S3P634). Further study will be needed to characterize its role in immune response in fish against bacterial infection. Up-regulated expression of these signaling proteins suggests that these proteins probably play a role in the defense of rainbow trout during *Y*. *ruckeri* infection. Interferon expression observed in this study suggests that the interferon signaling pathway is activated during *Y*. *ruckeri*-infection of rainbow trout.

In head kidney, most immune-related proteins such as c-type lectin-like receptors: A (2.3 to 4.8 fold) and B (2.0 to 4.3 fold) and precerebellin-like protein (2.5 to 10.8 fold) were significantly induced at 3 and 9 dpe in response to *Y*. *ruckeri* strains. C-type lectin-like receptors are important pathogen pattern recognition molecules that recognize carbohydrate structures. The up-regulation of C-type lectin domain family 4 (6.4 fold or 19.5 fold) was reported in the gill of Atlantic salmon in response to *Y*. *ruckeri* at 72 hours when assessed using microarray and real-time PCR^42^. The precerebellin-like protein has 46% similarity to the globular region of the C1q B chain of human and has been suggested to act as a part of the acute phase response in rainbow trout^43^. Expression of the gene encoding the precerebellin-like protein was up-regulated in rainbow trout at 3 dpe during *Y*. *ruckeri* infection^44,45^.

Using protein-protein interaction network analysis, 31 proteins showed high connectivity to each other by using textmining, experiments, and databases (Fig. 4). In this connection, the purple lines in Fig. 4 represent experimental evidence of protein-protein-interaction (PPI) in carbon metabolism and ribosome network pathways. As we see in Table 2 and Supplementary Table S2 that the expression of metabolic, ribosome and phagosome proteins were up-regulated during *Y*. *ruckeri* infection and connected to each other (Fig. 4). These results indicate that carbon metabolism, ribosome and phagosome pathways are actively involved in rainbow trout during *Y*. *ruckeri* infection. Carbon metabolism and ribosome pathways may be attributed to providing increased energy to fish to control infection. However, the phagosome pathway provides defence mechanisms and enhances survival of fish during yersiniosis. These pathways might be important in understanding biological processes and represent pioneering work on protein function research. It should be kept in mind that these predicted network pathways were based on zebrafish database using automated textmining, high-throughput lab experiments and previous knowledge in databases, where we found 43 to 100% identify (Supplementary Table S3) with rainbow trout amino acid sequences. Most of the salmonid protein data has not been certified experimentally. And the predicted rainbow trout PPI network pathway is expected to become more reliable with increasing quantities of fish proteins and the availability of salmonid database from PPI databases. Thus, PPI network pathways deserve further study.

Tinsley *et al*.^4^ reported marked distinct antigenic differences in the lipopolysaccharide profile of biotype 1 and biotype 2 *Y*. *ruckeri* isolates using rabbit anti-*Y*. *ruckeri* serum. However, in our study, these O antigen differences did not create major tissue proteomic changes in infected rainbow trout as observed in organ proteomic analysis using a shotgun proteomic approach, where we found minor proteomic expression differences in lymphoid organs in response to biotype 1 and biotype 2-infected rainbow trout. Some proteins such as flotillin-2a and caveolin membrane-associate proteins involved in a number of cellular functions including signaling, endocytosis and interactions with the cytoskeleton^46^ were either up- or down-regulated in rainbow trout during biotype 1 and biotype 2 *Y*. *ruckeri* strains infection (Supplementary Table S2). This suggests that biotypes 1 and 2 strains induce minor proteome alterations in rainbow trout. This is consistent with our previous study, in which we noted only minor proteomic differences between biotypes 1 and 2 strains grown under iron-limited^13^ and normal culture conditions^14^.

In conclusion, we studied for the first time the global proteomic profiles of rainbow trout lymphoid organs in response to experimental infection with *Y*. *ruckeri* biotypes 1 and 2 strains. We found that *Y*. *ruckeri* modulates the expression of a wide range of lymphoid organ proteins of various functions. These proteins show a strong protein-protein interaction in spleen of infected rainbow trout, and predicted carbon metabolism, ribosome and phagosome pathways might be useful in understanding biological processes and further pioneering work on protein function research. Some immune-related proteins such as lysozyme C, thioredoxin, chemotaxin, precerebellin-like protein, cathepsin B, C type lectin B and tetraspanin were strongly up-regulated in infected rainbow trout, which are essential to defense mechanisms against *Y*. *ruckeri* infection, and add new insights into the antibacterial activities in rainbow trout. Our presented protein expression data together with previous transcriptome studies will contribute to an extrapolation of the responses of the immune system in rainbow trout during *Y*. *ruckeri* infection.

## Materials and Methods

### *Y. ruckeri* strains

Biotype 1 strain CSF007-82 and biotype 2 strain 7959-11 were used for experimental infection of fish. These strains were isolated from diseased rainbow trout and both were serotype O1. Strain CSF007-82 originated from the USA^47^ and 7959-11 originated from Austria^13^. Strains were cultured in tryptic soya broth at 20 °C with shaking overnight and enumerated as colony forming units (CFU) by the spread plate method on blood agar^6^.

### Fish maintenance

Specific pathogen free rainbow trout (mean length 15 ± 1 cm, mean weight 32 ± 1 gm) were purchased from a certified Austrian hatchery. Fish were maintained in recirculating de-chlorinated water at 19 ± 1 °C at our facility. Fish were fed once per day (1% biomass) with commercial pelleted feed (4 mm) and acclimatized for 4 weeks. Prior to the experiment, fish were sampled randomly (n = 10) and tested using standard methods to confirm pathogen-free status.

### Fish experiment

Prior to infection, fish were distributed among 9 aquaria with 18 fish per aquarium. The following treatments were done in triplicate: (1) infection with CSF007-82, (2) infection with 7959-11, and (3) mock infection by exposure to diluted tryptic soya broth. Infections were initiated by immersion exposure consisting of 2 × 10^6^ CFU/mL of the bacterial strain in question for 2 hours in 30 L static oxygen saturated water. After 2 hours water flow was restored to each aquaria and fish were maintained under flow-through conditions at a 12 hours light and 12 hours dark cycle in aerated dechlorinated water (3 L per minute) at 19 ± 1 °C and fed daily (1% biomass). Mortality was monitored daily, and dead fish were removed immediately from the aquaria. Bacterial samples from the head kidney from all dead fish were taken and streaked on blood agar plates to confirm the cause of death. Mortality of fish was only considered to be caused by *Y*. *ruckeri* if the bacteria were recovered as a pure culture from the head kidney and confirmed using the MONO-Yr kit (BIONOR AS). No dead or moribund fish were sampled for the proteomic expression study. Nine fish (3 fish per aquarium) from the test and control groups were anaesthetised with 100 mg/L MS-222. Samples of individual organs (head kidney and spleen) were taken aseptically at 3, 9 and 28 dpe. The organs were washed three times with sterile phosphate-buffered saline containing a cocktail of mammalian protease inhibitors (Sigma). Each organ was divided into two portions, one snap-frozen in liquid nitrogen for proteomic study, and one fixed in RNA*Later* (Sigma) for molecular study and stored at −80 °C for further processing.

### Protein extraction

Equal amounts (40 mg) of each organ were pooled in 3 groups containing 3, 3 and 3 individual biological replicates, resulting in 3 pools to minimize the effects of fish-to-fish variation. Three pools for each organ of 9 individual biological replicates at 3, 9 and 28 dpe were prepared for three experimental groups (CSF007-82-infected, 7959-11-infected and control) for protein extraction and RNA purification described below. Therefore, each experimental group included a total of nine pools for each organ (in total 27 samples for each experimental group). Organs were ground into a fine powder using a mortar and pestle in liquid nitrogen. Ground powder (60 mg) of each sample was then resuspended in 1 mL precooled denaturing lysis buffer (7 M urea, 2 M thiourea, 4% CHAPS and 1% DTT) containing mammalian protease inhibitor cocktail (Sigma). The samples were incubated in an Eppendorf Thermomixer Comfort with shaking at 1200 rpm for 30 minutes at room temperature. Samples were then disrupted by sonication on ice for 10 cycles of 10 seconds pulse-on and 30 seconds pulse-off and incubated overnight at 4 °C in order to solubilize proteins. Cellular debris was removed by centrifugation at 12, 000 rpm for 20 minutes at 4 °C. Supernatants were collected and total protein concentration of each sample was determined colorimetrically with a NanoDrop 2000c spectrophotometer using a Pierce 660 nm Protein Assay.

### Protein digestion

Thirty micrograms protein of each sample was digested according to the standard enhanced filter-aided sample preparation protocol using Amicon Ultra 0.5 ml Ultracel 10 K centrifugal filters^48^. Briefly, after washing, proteins were reduced with dithiothreitol and alkylated with iodoacetamide. On-filter digestion was performed with Trypsin/Lys-C mix (Promega, Madison, WI) using 4% sodium deoxycholate for 14 hours at 37 °C. Digested peptides were recovered from the filter with three changes of 50 mM ammonium bicarbonate. Removal of sodium deoxycholate was achieved by phase transfer with ethyl acetate according to Erde *et al*.^48^. Extracted peptides were dried down in a vacuum concentrator (Eppendorf, Germany) and redissolved in 0.1% aqueous trifluoroacetic acid prior to LC-MS injection (3 µg protein absolute/injection). All samples were spiked with standardized indexed retention time reference peptides (iRT-Kit, Switzerland) for facilitation of retention time alignment.

### Micro LC-QTOF Mass Spectrometry for data-dependent acquisition

Peptides were separated on an Eksigent NanoLC 425 system using a microflow pump module (Sciex). Sample pre-concentration and desalting were accomplished with a 5 mm YMC-Triart C18 precolumn (500 µm inner diameter, 3 µm particle size, and 12 nm pore size). For sample loading and desalting ultra-pure LC-MS grade H_2_O with 0.1% formic acid was used as a mobile phase with a flow rate of 10 µL/min.

Separation of peptides was performed on a 15 cm YMC-Triart C18 column (300 µm inner diameter, 3 µm particle size, and 12 nm pore size) with a flow rate of 5 µL/min. The gradient started with 3% B (ACN with 0.1% FA) and increased in two steps to 25% B (68 min) and 35% (73 min). It was followed by a washing step with 80% B. Mobile Phase A consisted of ultra-pure H_2_O with 0.1% formic acid. For mass spectrometric analysis, the LC was directly coupled to a high resolution quadrupole time of flight mass spectrometer (Triple TOF 5600+, Sciex).

For information dependent data acquisition (IDA runs) MS1 spectra were collected in the range of 400–1250 m/z for 250 ms. The 40 most intense precursors with charge state 2–4, which exceeded 150 counts per second, were selected for fragmentation. MS2 spectra were collected in the range of 200–1500 m/z for 50 ms. Precursor ions were dynamically excluded from reselection for 13 seconds. The HPLC system was operated by Eksigent Control Software version 4.2 (Sciex) and the MS by Analyst Software 1.7.1 (Sciex).

### Data processing

Acquired raw data were processed with ProteinPilot Software version 5.0 (Sciex, USA) for re-calibration and database searches. The database consisted of UniProt entries of following taxonomies: *Oncorhynchus* (taxonomy id: 8016; entries: 51947), *Salmo salar* (taxonomy id: 8030; entries: 10025) and *Yersinia ruckeri* (taxonomy id: 29486; entries: 5300) [Released 2016_07] as well as cRAP (common Repository of Adventitious Proteins, downloaded: ftp://ftp.thegpm.org/fasta/cRAP/crap.fasta). Mass tolerance in MS mode was set with 0.05 and 0.1 Da in MSMS mode for the rapid recalibration search, and 0.0011 Da in MS and 0.01 Da in MSMS mode for the final search. The following sample parameters were applied: trypsin digestion, cysteine alkylation set to iodoacetamide, search effort set to rapid ID. False discovery rate analysis (FDR) was performed using the integrated tools in ProteinPilot and was set to <1% on protein peptide as well as on protein level.

### Quantification applying data-independent acquisition

IDA identification results were used to create the SWATH ion library with the MS/MS (ALL) with SWATH Acquisition MicroApp 2.0 in PeakView 2.2 (both Sciex). Peptides were chosen based on a FDR rate <1%, excluding shared and modified peptides. Up to 6 peptides per protein and up to 6 transitions per peptide were selected by software. Calculation of peak areas of SWATH samples after retention time alignment and normalization using total area sums was performed with MarkerView 1.2.1 (Sciex). Resulting protein lists were then used for visualization of data after PCA in the form of loadings plots and score plots to get a first impression of the overall data structure, and to assess variability between technical and biological replicates.

### Statistical analysis

To determine differentially expressed proteins in the infected organs, a statistical evaluation was performed in R programming language^49^. cRAP proteins were removed from the Marker View raw protein list before further processing. Raw peak areas after normalization to total area sums were log_2_-transformed to approach normal distribution. On a logarithmic scale, technical replicates were aggregated by arithmetic mean before application of statistical tests. This procedure is equivalent to the application of a hierarchical model in the subsequent ANOVA, as the same number of technical replicates was measured per biological replicate.

For each organ, differential expression of proteins was assessed using one-way ANOVA for each protein comparing all three conditions (control group, CSF007-82 and 7959-11) within each time point. To adjust for multiple testing, the method of Benjamini and Hochberg^50^ was used to control the FDR. Differences were considered significant if adjusted *p*-values were smaller than the significance level of *α* = 0.05. For those proteins, Tukey’s honest significant difference (HSD) method was applied as a post hoc test to assess the significance of the pairwise comparisons. Protein expression was considered differential if the adjusted *p*-value was below *α* and the absolute fold change was at least two (fold change <−2 or >+2).

### GO annotation

The software tool for rapid annotation of proteins (STRAP software version 1.5), UniProtKB database, AgBase-Goanna^51^ and PANTHER Classification System^52^ were used for classification of biological process, molecular function and pathway of differentially expressed proteins.

### Protein–protein interaction analysis

To determine the protein–protein interaction network of expressed proteins, amino acid sequences of identified 38 up-regulated proteins excluding uncharacterized proteins were blasted against zebrafish (*Danio rerio*) by using STRING software^53^. Representation of the protein-protein network was analyzed at confidence score 0.15 in the textmining, experiment and database interactions. Details of protein abbreviation and amino acid sequence identity are described in Supplementary Table S3.

### Quantitative real time PCR of selected proteins

Quantitative real time PCR was performed to examine the transcriptional regulation of differentially expressed proteins. Seven proteins: three differentially expressed (lysozyme C II, chemotaxin and protein S100) in both organs and four proteins (thioredoxin, p40phox, MHC I and beta-actin) only up-regulated in spleen were selected for analysis. The genes encoding these proteins were selected and qPCR primers were designed (Supplementary Table S4) using NCBI Primer-BLAST software (https://www.ncbi.nlm.nih.gov/tools/primer-blast/).

Total RNA was extracted from infected and control samples parallel with protein samples (described above) using an RNeasy Mini Kit (Qiagen) and included an on-column DNase digestion step. cDNA was synthesized using an iScript cDNA Synthesis Kit (Bio-Rad) with 500 ng total RNA. The cDNA samples of infected and control samples at different time points (in total 27 head kidney and 27 spleen samples) were subjected to qPCR with technical replicates using selected gene primers. qPCRs were performed in a final volume of 20 μL, which contained 4 μL of 1:10 fold diluted cDNA, 0.4 μM of each primer, 1X SsoAdvanced™ Universal SYBR Green Supermix (Bio-Rad) and DEPC-treated sterile distilled water (Carl Roth). After 5 minutes of cDNA denaturation at 95 °C, 37 cycles were performed at 95 °C for 30 seconds, 53–58 °C for 30 seconds and 72 °C for 30 seconds in a CFX96 Touch Real-Time PCR detection system (Bio-Rad). The relative quantity values of the test samples were normalized to a reference gene, elongation factor alpha 1^54^ using the CFX Manager Software in normalized expression mode (∆∆Cq) and differences in relative transcription were statistically analysed using *t*-tests. The relative quantities of samples were normalized with reference gene. Relative expression levels of target transcripts were analysed at each time point using a linear mixed effect model. Adjustment for multiple comparisons was performed using SIDAK’s procedure. The differences between infected and control groups at each single time point were analysed using t-tests for independent samples with Bonferroni α-correction. For all statistical tests, a *p-*value < 0.05 was regarded as significant. All statistical analyses were conducted with IBM SPSS version-23 software.

### Ethics statement

This study was approved by the institutional ethics committee of the University of Veterinary Medicine Vienna and the national authority, according to §26 of the Austrian Law for Animal Experiments, Tierversuchsgesetz 2012 under approval number BMWFW-68.205/0041-WF/V/3b/2015.

## Electronic supplementary material

A complete list of differentially expressed proteins of head kidney of rainbow trout in response to Yersinia ruckeri strains

A complete list of differentially expressed proteins of spleen of rainbow trout in response to Yersinia ruckeri strains.

List of protein abbreviation used in the protein-protein interaction network.

List of quantitative real-time PCR primers.

## Data Availability

Shotgun proteomics data generated during the current study have been deposited in the ProteomeXchange Consortium (http://www.proteomexchange.org/) via the PRIDE partner repository^55^ with the dataset identifiers PXD008473 (head kidney) and PXD008478 (spleen).
