# Author Correction: Proteome analysis reveals a role of rainbow trout lymphoid organs during *Yersinia ruckeri* infection process

**DOI:** 10.1038/s41598-018-33154-y

**Published:** 2018-10-12

**Authors:** Gokhlesh Kumar, Karin Hummel, Katharina Noebauer, Timothy J. Welch, Ebrahim Razzazi-Fazeli, Mansour El-Matbouli

**Affiliations:** 1 Clinical Division of Fish Medicine, University of Veterinary Medicine, Vienna, Austria; 2 VetCore Facility for Research/Proteomics Unit, University of Veterinary Medicine, Vienna, Austria; 3 National Center for Cool and Cold Water Aquaculture, Kearneysville, USA

Correction to: *Scientific Reports* 10.1038/s41598-018-31982-6, published online 18 September 2018

The original version of this Article contained an incorrect version of the Supplementary Information file “List of quantitative real-time PCR primers.” This error has now been corrected in the HTML version of the Article; the PDF version was correct from the time of publication.

