# Supplementary material for: Proteome analysis reveals a role of rainbow trout lymphoid organs during Yersinia ruckeri infection process
Source: Sci Rep. 2018 Sep 18;8:13998. doi: 10.1038/s41598-018-31982-6 (PMC6143608; doi:10.1038/s41598-018-31982-6)
Supplement: Supplementary file 1 — A complete list of differentially expressed proteins of head kidney of rainbow trout in response to Yersinia ruckeri strains [file 41598_2018_31982_MOESM1_ESM.doc]

**Proteome analysis reveals a role of rainbow trout lymphoid organs** **during *Yersinia ruckeri* infection process**

Gokhlesh Kumar^1^*, Karin Hummel^2^, Katharina Noebauer^2^, Timothy J Welch^3^, Ebrahim Razzazi-Fazeli^2^ & Mansour El-Matbouli^1^

# ^1^Clinical Division of Fish Medicine, University of Veterinary Medicine, Vienna, Austria

^2^VetCore Facility for Research / Proteomics Unit, University of Veterinary Medicine, Vienna, Austria

^3^National Center for Cool and Cold Water Aquaculture, Kearneysville, USA

*Corresponding Author

**Supplementary Table S1:** A complete list of differentially expressed proteins of head kidney of rainbow trout in response to *Yersinia ruckeri* strains. Fold change (infected vs control) was statistically analysed in *Y. ruckeri* CSF007-82 (biotype 1) and 7959-11 (biotype 2) infected and control rainbow trout samples (n = 27). *denotes statistically significant difference according to both ANOVA and post hoc Tukey’s HSD with FDR-adjusted p-value <0.05 and fold change < −2 or > +2.

| **Accession** | **Protein** | **No. of quantified peptides** | **FDR-adjusted p-value ANOVA (3 dpe)** | **FDR-adjusted p-value ANOVA (9 dpe)** | **FDR-adjusted p-value ANOVA (28 dpe)** | **Head kidney control in response to strain** | **Fold change 3 dpe** | **Fold change 9 dpe** | **Fold change 28 dpe** |
| --- | --- | --- | --- | --- | --- | --- | --- | --- | --- |
| LYSC2_ONCMY | Lysozyme C II | 6 | 0.11 | 0.02 | 0.75 | CSF007-82 | 2.2 | **4.8*** | 2.2 |
|  |  |  |  |  |  | 7959-11 | 2.0 | **4.8*** | 1.2 |
| W8W0Y8_ONCMY | Glutathione peroxidase | 6 | 0.07 | 0.03 | 0.99 | CSF007-82 | -1.9 | **-2.6*** | -1.3 |
|  |  |  |  |  |  | 7959-11 | -2.5 | -1.4 | -1.1 |
| Q9PT14_ONCMY | Precerebellin-like protein | 5 | 0.08 | 0.03 | 0.83 | CSF007-82 | 2.9 | **10.8*** | 2.9 |
|  |  |  |  |  |  | 7959-11 | 2.5 | **6.4*** | 1.9 |
| Q9DFJ1_ONCMY | Chemotaxin (Fragment) | 5 | 0.02 | 0.15 | 0.83 | CSF007-82 | **4.1*** | 2.9 | 1.1 |
|  |  |  |  |  |  | 7959-11 | **2.7*** | 2.1 | -1.3 |
| Q68S98_SALSA | C type lectin receptor A | 2 | 0.01 | 0.01 | 0.99 | CSF007-82 | **2.6*** | **4.8*** | 1.4 |
|  |  |  |  |  |  | 7959-11 | **2.3*** | **3.6*** | 1.2 |
| C0KIP4_ONCMY | C type lectin receptor B | 4 | 0.04 | 0.03 | 0.83 | CSF007-82 | **2.1*** | **4.3*** | 2.1 |
|  |  |  |  |  |  | 7959-11 | **2.0*** | **2.8*** | 1.5 |
| C0HBD4_SALSA | Interferon-induced guanylate-binding protein 1 | 2 | 0.84 | 0.03 | 0.83 | CSF007-82 | 1.1 | **2.4*** | 1.4 |
|  |  |  |  |  |  | 7959-11 | 1.1 | 1.9 | 1.5 |
| Q5DVP5_ONCMY | Interleukin-16 (Fragment) | 2 | 0.03 | 0.05 | 0.83 | CSF007-82 | **2.1*** | 2.1 | 2.2 |
|  |  |  |  |  |  | 7959-11 | **2.3*** | 2.3 | 1.6 |
| C0H8U7_SALSA | Cold-inducible RNA-binding protein | 3 | 0.02 | 0.29 | 0.99 | CSF007-82 | 1.9 | 1.6 | 1.0 |
|  |  |  |  |  |  | 7959-11 | **2.1*** | 1.4 | 1.2 |
| B5DG79_SALSA | RNA-binding protein 4B | 2 | 0.03 | 0.10 | 0.55 | CSF007-82 | **2.6*** | 2.3 | 1.6 |
|  |  |  |  |  |  | 7959-11 | **2.7*** | 1.7 | 1.1 |
| A0A060XEG1_ONCMY | Hemopexin | 5 | 0.31 | 0.04 | 0.99 | CSF007-82 | 1.3 | **2.8*** | 1.6 |
|  |  |  |  |  |  | 7959-11 | 1.3 | **2.1*** | 1.3 |
| B5XDZ7_SALSA | Catechol-O-methyltransferase domain-containing protein 1 | 5 | 0.04 | 0.03 | 0.83 | CSF007-82 | **2.4*** | **6.6*** | 2.3 |
|  |  |  |  |  |  | 7959-11 | **2.2*** | **3.3*** | 1.4 |
| B5RI95_SALSA | Cold-inducible RNA-binding protein | 4 | 0.03 | 0.26 | 0.83 | CSF007-82 | 1.8 | 1.6 | 1.4 |
|  |  |  |  |  |  | 7959-11 | **2.1*** | 1.3 | 1.2 |
| B5X215_SALSA | Ribonucleoside-diphosphate reductase subunit M2 | 4 | 0.03 | 0.05 | 0.99 | CSF007-82 | **2.3*** | 2.2 | 1.1 |
|  |  |  |  |  |  | 7959-11 | **2.2*** | 1.5 | -1.3 |
| A0A060X1N2_ONCMY | DNA helicase | 2 | 0.09 | 0.03 | 0.94 | CSF007-82 | 1.7 | **3.3*** | 1.4 |
|  |  |  |  |  |  | 7959-11 | 1.8 | **2.3*** | -1.4 |
| I3WWD7_ONCMY | Plasminogen activator inhibitor 1 | 2 | 0.04 | 0.53 | 0.99 | CSF007-82 | **3.2*** | 1.4 | 1.2 |
|  |  |  |  |  |  | 7959-11 | **2.9*** | 1.3 | -1.1 |
| Q5NKG0_ONCMY | Chitinase | 5 | 0.15 | 0.04 | 0.65 | CSF007-82 | 1.4 | **2.1*** | 1.5 |
|  |  |  |  |  |  | 7959-11 | 1.6 | 1.9 | 1.4 |
| C1BHS7_ONCMY | Protein S100 | 2 | 0.05 | 0.04 | 0.99 | CSF007-82 | -1.4 | **-2.2*** | -1.1 |
|  |  |  |  |  |  | 7959-11 | -1.4 | -1.6 | 1.1 |
| Q90YG8_ONCMY | Putative ribosomal protein L27A protein (Fragment) | 3 | 0.03 | 0.06 | 0.99 | CSF007-82 | **2.1*** | 1.9 | 1.2 |
|  |  |  |  |  |  | 7959-11 | **2.0*** | 1.4 | 1.3 |
| A0A060XGG8_ONCMY | Uncharacterized protein | 3 | 0.02 | 0.38 | 0.75 | CSF007-82 | **2.1*** | 1.5 | 2.6 |
|  |  |  |  |  |  | 7959-11 | 1.9 | 1.3 | -1.0 |
| A0A060YYG8_ONCMY | Uncharacterized protein | 4 | 0.02 | 0.49 | 0.83 | CSF007-82 | 1.9 | 1.5 | 1.1 |
|  |  |  |  |  |  | 7959-11 | **2.1*** | 1.3 | 1.2 |
| A0A060VQI0_ONCMY | Uncharacterized protein | 3 | 0.19 | 0.04 | 0.99 | CSF007-82 | 1.8 | **4.0*** | 1.0 |
|  |  |  |  |  |  | 7959-11 | 1.9 | **2.8*** | -1.1 |
| A0A060XFB5_ONCMY | Uncharacterized protein | 2 | 0.04 | 0.43 | 0.83 | CSF007-82 | **2.1*** | 1.8 | 1.7 |
|  |  |  |  |  |  | 7959-11 | **2.2*** | 1.3 | 1.2 |
| A0A060XAN2_ONCMY | Uncharacterized protein | 2 | 0.01 | 0.03 | 0.99 | CSF007-82 | **2.2*** | **2.6*** | 1.4 |
|  |  |  |  |  |  | 7959-11 | **2.3*** | 2.0 | 1.4 |
| A0A060XP00_ONCMY | Uncharacterized protein | 2 | 0.03 | 0.04 | 0.95 | CSF007-82 | **3.0*** | 2.0 | 1.7 |
|  |  |  |  |  |  | 7959-11 | **3.4*** | 1.7 | 1.1 |
| A0A060XJJ4_ONCMY | Uncharacterized protein | 6 | 0.02 | 0.06 | 0.99 | CSF007-82 | **2.3*** | 1.7 | 1.2 |
|  |  |  |  |  |  | 7959-11 | **2.3*** | 1.5 | 1.1 |
| A0A060XZX9_ONCMY | Uncharacterized protein | 3 | 0.05 | 0.07 | 0.99 | CSF007-82 | **2.0*** | 1.8 | 1.3 |
|  |  |  |  |  |  | 7959-11 | **2.0*** | 1.7 | 1.0 |
| A0A060YPC2_ONCMY | Uncharacterized protein | 5 | 0.03 | 0.39 | 0.99 | CSF007-82 | **3.7*** | 2.1 | 1.2 |
|  |  |  |  |  |  | 7959-11 | **3.1*** | 1.6 | -1.0 |
| A0A060YZR6_ONCMY | Uncharacterized protein | 6 | 0.01 | 0.10 | 0.83 | CSF007-82 | **3.8*** | 2.3 | 1.2 |
|  |  |  |  |  |  | 7959-11 | **2.8*** | 2.0 | -1.2 |
| A0A060Y8C2_ONCMY | Uncharacterized protein | 3 | 0.00 | 0.37 | 0.83 | CSF007-82 | **3.0*** | 1.7 | 1.8 |
|  |  |  |  |  |  | 7959-11 | **2.9*** | 1.3 | 1.4 |
| A0A060W3L6_ONCMY | Uncharacterized protein | 5 | 0.02 | 0.46 | 0.99 | CSF007-82 | 1.9 | 1.4 | 1.2 |
|  |  |  |  |  |  | 7959-11 | **2.0*** | 1.3 | 1.1 |
| A0A060XKK4_ONCMY | Uncharacterized protein | 5 | 0.03 | 0.04 | 0.89 | CSF007-82 | **7.4*** | **13.3*** | 5.8 |
|  |  |  |  |  |  | 7959-11 | **7.1*** | **6.9*** | 3.2 |
| A0A060YAR2_ONCMY | Uncharacterized protein | 3 | 0.46 | 0.04 | 0.99 | CSF007-82 | 1.2 | **4.8*** | 1.1 |
|  |  |  |  |  |  | 7959-11 | 1.5 | **3.9*** | 1.3 |
| A0A060Y0R7_ONCMY | Uncharacterized protein | 3 | 0.12 | 0.04 | 0.85 | CSF007-82 | 1.3 | **2.7*** | -1.6 |
|  |  |  |  |  |  | 7959-11 | -1.0 | **2.1*** | 1.0 |
