# Supplementary material for: Proteome analysis reveals a role of rainbow trout lymphoid organs during Yersinia ruckeri infection process
Source: Sci Rep. 2018 Sep 18;8:13998. doi: 10.1038/s41598-018-31982-6 (PMC6143608; doi:10.1038/s41598-018-31982-6)
Supplement: Supplementary file 2 — A complete list of differentially expressed proteins of spleen of rainbow trout in response to Yersinia ruckeri strains. [file 41598_2018_31982_MOESM2_ESM.doc]

**Proteome analysis reveals a role of rainbow trout lymphoid organs** **during *Yersinia ruckeri* infection process**

Gokhlesh Kumar^1^*. Karin Hummel^2^. Katharina Noebauer^2^. Timothy J Welch^3^. Ebrahim Razzazi-Fazeli^2^ & Mansour El-Matbouli^1^

# ^1^Clinical Division of Fish Medicine. University of Veterinary Medicine. Vienna. Austria

^2^VetCore Facility for Research / Proteomics Unit. University of Veterinary Medicine. Vienna. Austria

^3^National Center for Cool and Cold Water Aquaculture. Kearneysville. USA

*Corresponding Author

**Supplementary Table S2:** A complete list of differentially expressed proteins of spleen of rainbow trout in response to *Yersinia ruckeri* strains. Fold change (infected vs control) was statistically analysed in *Y. ruckeri* CSF007-82 (biotype 1) and 7959-11 (biotype 2) infected and control rainbow trout samples (n = 27). *denotes statistically significant difference according to both ANOVA and post hoc Tukey’s HSD with FDR-adjusted p-value <0.05 and fold change < −2 or > +2.

| **Accession** | **Protein** | **No. of quantified peptides** | **FDR-adjusted p-value ANOVA (3 dpe)** | **FDR-adjusted p-value ANOVA (9 dpe)** | **FDR-adjusted p-value ANOVA (28 dpe)** | **Spleen control in response to strain** | **Fold change 3 dpe** | **Fold change 9 dpe** | **Fold change 28 dpe** |
| --- | --- | --- | --- | --- | --- | --- | --- | --- | --- |
| LYSC2_ONCMY | Lysozyme C II | 6 | 0.04 | 0.16 | 0.42 | CSF007-82 | **4.6*** | 11.8 | 3.7 |
|  |  |  |  |  |  | 7959-11 | **3.0*** | 6.6 | 3.5 |
| W8W0Y8_ONCMY | Glutathione peroxidase | 5 | 0.03 | 0.05 | 0.12 | CSF007-82 | 1.9 | 1.3 | 1.6 |
|  |  |  |  |  |  | 7959-11 | **2.2*** | 1.9 | 1.4 |
| Q60FB5_ONCMY | NADPH oxidase cytosolic protein p67phox | 4 | 0.03 | 0.22 | 0.76 | CSF007-82 | **2.7*** | 2.2 | -1.3 |
|  |  |  |  |  |  | 7959-11 | **3.1*** | 2.1 | -1.4 |
| Q60FB6_ONCMY | NADPH oxidase cytosolic protein p40phox | 6 | 0.03 | 0.14 | 0.05 | CSF007-82 | **3.0*** | 2.6 | 1.5 |
|  |  |  |  |  |  | 7959-11 | **3.0*** | 2.2 | 1.3 |
| C1BHL9_ONCMY | Ras-related C3 botulinum toxin substrate 2 | 2 | 0.00 | 0.01 | 0.14 | CSF007-82 | **7.3*** | **5.4*** | 3.6 |
|  |  |  |  |  |  | 7959-11 | **5.2*** | **4.9*** | 3.4 |
| C1BFF6_ONCMY | Thioredoxin | 4 | 0.03 | 0.03 | 0.10 | CSF007-82 | 1.8 | **2.3*** | 2.0 |
|  |  |  |  |  |  | 7959-11 | 1.6 | 2.0 | 1.8 |
| C1BH85_ONCMY | Thioredoxin | 5 | 0.02 | 0.07 | 0.10 | CSF007-82 | **3.3*** | 4.8 | 3.6 |
|  |  |  |  |  |  | 7959-11 | **2.8*** | 2.6 | 3.1 |
| B5X4P4_SALSA | Cathepsin B | 2 | 0.12 | 0.02 | 0.05 | CSF007-82 | 2.3 | **3.8*** | **2.6*** |
|  |  |  |  |  |  | 7959-11 | 1.5 | **2.9*** | **2.6*** |
| Q9DFJ1_ONCMY | Chemotaxin (Fragment) | 5 | 0.03 | 0.05 | 0.63 | CSF007-82 | **4.9*** | 3.6 | 1.5 |
|  |  |  |  |  |  | 7959-11 | **4.2*** | 3.0 | 1.3 |
| Q92004_ONCMY | Beta-2-microglobulin | 4 | 0.13 | 0.05 | 0.35 | CSF007-82 | 2.2 | **4.1*** | 2.0 |
|  |  |  |  |  |  | 7959-11 | 1.7 | **3.0*** | 2.1 |
| A0A060VM33_ONCMY | Metalloendopeptidase | 6 | 0.05 | 0.35 | 0.05 | CSF007-82 | 4.0 | -1.3 | **3.1*** |
|  |  |  |  |  |  | 7959-11 | 4.7 | 1.5 | 1.7 |
| C0HBR8_SALSA | Metalloreductase STEAP4 | 2 | 0.35 | 0.02 | 0.78 | CSF007-82 | 2.2 | **3.7*** | 1.2 |
|  |  |  |  |  |  | 7959-11 | 2.6 | **2.2*** | 1.4 |
| B5X1B5_SALSA | Alpha-enolase | 2 | 0.01 | 0.01 | 0.05 | CSF007-82 | **3.1*** | **3.0*** | **2.4*** |
|  |  |  |  |  |  | 7959-11 | **3.2*** | **2.9*** | 1.8 |
| A0A060VVF4_ONCMY | Transgelin | 2 | 0.16 | 0.03 | 0.07 | CSF007-82 | 2.9 | **2.4*** | 2.5 |
|  |  |  |  |  |  | 7959-11 | 2.5 | 1.6 | 1.8 |
| B9EM17_SALSA | Transaldolase | 2 | 0.04 | 0.35 | 0.40 | CSF007-82 | **2.5*** | 1.6 | 1.3 |
|  |  |  |  |  |  | 7959-11 | **2.6*** | 1.6 | 1.3 |
| A0A060X145_ONCMY | Tetraspanin | 2 | 0.02 | 0.02 | 0.04 | CSF007-82 | **4.0*** | **4.5*** | **3.7*** |
|  |  |  |  |  |  | 7959-11 | **2.9*** | **5.2*** | **3.2*** |
| C0PUI9_SALSA | Fructose-1,6-bisphosphatase 1 (Fragment) | 6 | 0.03 | 0.35 | 0.04 | CSF007-82 | **3.6*** | 1.5 | **2.5*** |
|  |  |  |  |  |  | 7959-11 | **4.0*** | 1.9 | 1.7 |
| A0A060XEX6_ONCMY | Glucose-6-phosphate 1-dehydrogenase | 6 | 0.05 | 0.30 | 0.40 | CSF007-82 | **2.3*** | 1.5 | 1.3 |
|  |  |  |  |  |  | 7959-11 | **3.0*** | 1.4 | 1.0 |
| B5X4R9_SALSA | 6-phosphofructo-2-kinase/fructose-2,6-biphosphatase 4 | 2 | 0.04 | 0.71 | 0.93 | CSF007-82 | 1.5 | 1.1 | 1.0 |
|  |  |  |  |  |  | 7959-11 | **2.0*** | 1.2 | 1.1 |
| C0H8V3_SALSA | Pyruvate kinase | 3 | 0.23 | 0.03 | 0.66 | CSF007-82 | 1.8 | 1.8 | 1.3 |
|  |  |  |  |  |  | 7959-11 | 1.9 | **2.2*** | 1.2 |
| A0A060Y7U5_ONCMY | Aminomethyltransferase | 2 | 0.89 | 0.37 | 0.01 | CSF007-82 | 1.5 | -1.5 | **17.8*** |
|  |  |  |  |  |  | 7959-11 | 2.2 | 1.3 | **10.3*** |
| B5X1B2_SALSA | Phosphoacetylglucosamine mutase | 3 | 0.66 | 0.03 | 0.65 | CSF007-82 | 1.2 | **2.1*** | 1.1 |
|  |  |  |  |  |  | 7959-11 | 1.2 | 1.9 | 1.1 |
| B5X0W0_SALSA | Serine/threonine-protein phosphatase | 3 | 0.14 | 0.01 | 0.34 | CSF007-82 | 1.7 | 1.9 | 1.4 |
|  |  |  |  |  |  | 7959-11 | 1.5 | **2.0*** | 1.2 |
| C1BHY0_ONCMY | Epididymal secretory protein E1 | 4 | 0.10 | 0.04 | 0.57 | CSF007-82 | 2.3 | **4.9*** | 1.6 |
|  |  |  |  |  |  | 7959-11 | 1.8 | **3.0*** | 1.8 |
| C1BEX7_ONCMY | Hemoglobin subunit alpha-4 | 2 | 0.30 | 0.03 | 0.09 | CSF007-82 | 2.3 | **2.7*** | 3.0 |
|  |  |  |  |  |  | 7959-11 | 1.5 | 1.7 | 2.3 |
| B5XAH0_SALSA | Fatty acid-binding protein intestinal | 5 | 0.02 | 0.29 | 0.87 | CSF007-82 | -1.3 | 1.2 | -1.1 |
|  |  |  |  |  |  | 7959-11 | **-2.9*** | -1.4 | 1.1 |
| C0HBS0_SALSA | ADP/ATP translocase 2 | 5 | 0.66 | 0.03 | 0.35 | CSF007-82 | 1.3 | **2.0*** | 1.0 |
|  |  |  |  |  |  | 7959-11 | 1.3 | 1.8 | 1.2 |
| B9ENC0_SALSA | Cellular nucleic acid-binding protein | 5 | 0.02 | 0.00 | 0.03 | CSF007-82 | **5.4*** | **7.2*** | **5.7*** |
|  |  |  |  |  |  | 7959-11 | **4.5*** | **7.1*** | **4.3*** |
| F8RP06_ONCMA | Ribosomal protein S5 | 5 | 0.01 | 0.00 | 0.05 | CSF007-82 | **3.6*** | **3.7*** | **3*** |
|  |  |  |  |  |  | 7959-11 | **3.2*** | **3.2*** | **2.7*** |
| B5DGY1_SALSA | Ribosomal protein S27-3 | 2 | 0.39 | 0.03 | 0.52 | CSF007-82 | 1.8 | **2.7*** | 1.7 |
|  |  |  |  |  |  | 7959-11 | 1.2 | 1.8 | 1.6 |
| C1BHQ0_ONCMY | 60S ribosomal protein L36 | 4 | 0.11 | 0.03 | 0.35 | CSF007-82 | 1.5 | **2.2*** | 1.3 |
|  |  |  |  |  |  | 7959-11 | 1.3 | 1.8 | 1.4 |
| B5DGG8_SALSA | 40S ribosomal protein S12 | 3 | 0.02 | 0.03 | 0.09 | CSF007-82 | **2.1*** | **2.7*** | 1.7 |
|  |  |  |  |  |  | 7959-11 | 1.6 | 2.0 | 1.7 |
| B9EL24_SALSA | Small nuclear ribonucleoprotein F | 2 | 0.02 | 0.03 | 0.02 | CSF007-82 | **2.1*** | **2.7*** | **2.4*** |
|  |  |  |  |  |  | 7959-11 | **2.2*** | **2.6*** | **2.8*** |
| Q8UUJ3_ONCKE | Type I collagen alpha 2 chain (Fragment) | 6 | 0.04 | 0.10 | 0.18 | CSF007-82 | -1.7 | -2.0 | -2.1 |
|  |  |  |  |  |  | 7959-11 | **-2.0*** | -1.7 | -1.5 |
| B5X1G4_SALSA | Cysteinyl-tRNA synthetase, cytoplasmic | 6 | 0.55 | 0.03 | 0.48 | CSF007-82 | 1.5 | **3.5*** | 1.2 |
|  |  |  |  |  |  | 7959-11 | 1.8 | **2.7*** | 1.7 |
| C0HB50_SALSA | Probable ATP-dependent RNA helicase DDX5 | 5 | 0.03 | 0.05 | 0.79 | CSF007-82 | 1.9 | 2.3 | 1.1 |
|  |  |  |  |  |  | 7959-11 | **2.6*** | 2.0 | 1.2 |
| Q9I9G8_ONCMY | B-actin (Fragment) | 2 | 0.02 | 0.04 | 0.19 | CSF007-82 | **2.1*** | 1.9 | 1.6 |
|  |  |  |  |  |  | 7959-11 | **2.1*** | 1.8 | 1.5 |
| C1BH21_ONCMY | Dynein light chain 1, cytoplasmic | 3 | 0.06 | 0.01 | 0.04 | CSF007-82 | 2.0 | **2.9*** | **2.4*** |
|  |  |  |  |  |  | 7959-11 | 1.7 | **2.9*** | **2.1*** |
| B5X1V0_SALSA | Erythrocyte band 7 integral membrane protein | 2 | 0.47 | 0.02 | 0.82 | CSF007-82 | 1.2 | **2.9*** | 1.1 |
|  |  |  |  |  |  | 7959-11 | 1.5 | **2.6*** | 1.0 |
| C1BGJ3_ONCMY | Transmembrane emp24 domain-containing protein 7 | 2 | 0.05 | 0.02 | 0.33 | CSF007-82 | 2.9 | **2.6*** | 1.6 |
|  |  |  |  |  |  | 7959-11 | 2.5 | **2.2*** | 1.5 |
| K1C18_ONCMY | Keratin. type I cytoskeletal 18 | 6 | 0.02 | 0.10 | 0.34 | CSF007-82 | -1.4 | -1.1 | -1.6 |
|  |  |  |  |  |  | 7959-11 | **-2.0*** | -1.4 | -1.2 |
| A0A060Z9I8_ONCMY | Protein S100 | 3 | 0.01 | 0.07 | 0.53 | CSF007-82 | -1.7 | -1.4 | -1.5 |
|  |  |  |  |  |  | 7959-11 | **-3.2*** | -1.8 | -1.1 |
| C1BHS7_ONCMY | Protein S100 | 2 | 0.04 | 0.09 | 0.10 | CSF007-82 | -1.3 | -1.5 | -1.1 |
|  |  |  |  |  |  | 7959-11 | **-2.4*** | -1.4 | 1.2 |
| C1BEZ5_ONCMY | C6orf115 | 2 | 0.02 | 0.03 | 0.03 | CSF007-82 | **3.0*** | **4.0*** | **2.8*** |
|  |  |  |  |  |  | 7959-11 | **2.5*** | **2.5*** | **2.2*** |
| A0A060Y668_ONCMY | Caveolin | 2 | 0.68 | 0.69 | 0.01 | CSF007-82 | 1.4 | -1.8 | **-7.8*** |
|  |  |  |  |  |  | 7959-11 | -1.1 | -1.3 | 1.4 |
| B5X242_SALSA | Flotillin-2a | 3 | 0.90 | 0.03 | 0.52 | CSF007-82 | -1.2 | -1.2 | 1.2 |
|  |  |  |  |  |  | 7959-11 | -1.0 | **2.1*** | 1.5 |
| A0A060YAR2_ONCMY | Uncharacterized protein | 4 | 0.03 | 0.13 | 0.45 | CSF007-82 | **5.8*** | 7.7 | 1.6 |
|  |  |  |  |  |  | 7959-11 | **5.4*** | 5.3 | 2.5 |
| A0A060YML0_ONCMY | Uncharacterized protein | 4 | 0.13 | 0.03 | 0.17 | CSF007-82 | 2.9 | **2.7*** | 1.5 |
|  |  |  |  |  |  | 7959-11 | 2.1 | **2.3*** | 1.4 |
| A0A060YTX4_ONCMY | Uncharacterized protein (Fragment) | 2 | 0.14 | 0.03 | 0.19 | CSF007-82 | 1.9 | **4.7*** | 2.0 |
|  |  |  |  |  |  | 7959-11 | 1.3 | **2.7*** | 1.9 |
| A0A060YV33_ONCMY | Uncharacterized protein (Fragment) | 5 | 0.04 | 0.24 | 0.80 | CSF007-82 | **2.3*** | 1.0 | 1.4 |
|  |  |  |  |  |  | 7959-11 | **2.7*** | 1.5 | 1.0 |
| A0A060WK59_ONCMY | Uncharacterized protein | 4 | 0.55 | 0.05 | 0.65 | CSF007-82 | 2.3 | **2.5*** | 1.5 |
|  |  |  |  |  |  | 7959-11 | 2.0 | 1.6 | 1.2 |
| A0A060YMT8_ONCMY | Uncharacterized protein | 6 | 0.05 | 0.64 | 0.44 | CSF007-82 | **2.5*** | 1.2 | 1.5 |
|  |  |  |  |  |  | 7959-11 | **3.1*** | 1.7 | 1.3 |
| A0A060WLD1_ONCMY | Uncharacterized protein | 6 | 0.03 | 0.20 | 0.61 | CSF007-82 | **2.1*** | 2.4 | 1.4 |
|  |  |  |  |  |  | 7959-11 | **2.4*** | 1.5 | 1.3 |
| A0A060YHU0_ONCMY | Uncharacterized protein | 4 | 0.24 | 0.02 | 0.45 | CSF007-82 | 1.7 | **3.0*** | 1.4 |
|  |  |  |  |  |  | 7959-11 | 1.6 | **2.3*** | 1.4 |
| A0A060XQJ4_ONCMY | Uncharacterized protein | 3 | 0.03 | 0.06 | 0.76 | CSF007-82 | **2.1*** | 1.7 | 1.2 |
|  |  |  |  |  |  | 7959-11 | **2.3*** | 1.6 | 1.1 |
| A0A060Z016_ONCMY | Uncharacterized protein (Fragment) | 3 | 0.03 | 0.97 | 0.04 | CSF007-82 | **3.1*** | -1.3 | **2.0*** |
|  |  |  |  |  |  | 7959-11 | **3.2*** | -1.2 | 1.6 |
| A0A060YZE3_ONCMY | Uncharacterized protein | 2 | 0.05 | 0.02 | 0.78 | CSF007-82 | 3.6 | **4.4*** | 1.0 |
|  |  |  |  |  |  | 7959-11 | 2.2 | **2.5*** | -1.7 |
| A0A060Y8K0_ONCMY | Uncharacterized protein (Fragment) | 2 | 0.02 | 0.08 | 0.57 | CSF007-82 | **4.4*** | 5.8 | 1.4 |
|  |  |  |  |  |  | 7959-11 | **3.3*** | 2.5 | 1.6 |
| A0A060XSB4_ONCMY | Uncharacterized protein | 3 | 0.23 | 0.03 | 0.04 | CSF007-82 | 3.0 | **2.9*** | **4.4*** |
|  |  |  |  |  |  | 7959-11 | 2.1 | **2.3*** | **2.9*** |
| A0A060W2V1_ONCMY | Uncharacterized protein | 4 | 0.02 | 0.05 | 0.07 | CSF007-82 | **2.4*** | 2.1 | 2.1 |
|  |  |  |  |  |  | 7959-11 | **2.5*** | 1.8 | 1.9 |
| A0A060ZFF1_ONCMY | Uncharacterized protein | 2 | 0.13 | 0.03 | 0.18 | CSF007-82 | 1.8 | **3.1*** | 1.7 |
|  |  |  |  |  |  | 7959-11 | 2.6 | **2.4*** | 1.4 |
| A0A060XU19_ONCMY | Uncharacterized protein | 2 | 0.02 | 0.22 | 0.18 | CSF007-82 | **2.2*** | 2.1 | 2.1 |
|  |  |  |  |  |  | 7959-11 | **2.8*** | 2.1 | 1.9 |
| A0A060XFS7_ONCMY | Uncharacterized protein (Fragment) | 4 | 0.02 | 0.04 | 0.49 | CSF007-82 | **2.1*** | **2.3*** | 1.7 |
|  |  |  |  |  |  | 7959-11 | 1.8 | 1.9 | 3.7 |
| A0A060YG41_ONCMY | Uncharacterized protein | 2 | 0.08 | 0.03 | 0.23 | CSF007-82 | 3.2 | **4.9*** | 3.0 |
|  |  |  |  |  |  | 7959-11 | 1.9 | **3.3*** | 2.1 |
| A0A060ZAC8_ONCMY | Uncharacterized protein (Fragment) | 2 | 0.04 | 0.13 | 0.36 | CSF007-82 | **2.3*** | 1.5 | 1.8 |
|  |  |  |  |  |  | 7959-11 | **3.0*** | 1.8 | 1.3 |
| A0A060YMH1_ONCMY | Uncharacterized protein | 3 | 0.09 | 0.03 | 0.35 | CSF007-82 | 1.8 | **2.1*** | 1.5 |
|  |  |  |  |  |  | 7959-11 | 1.8 | **2.3*** | 1.4 |
| A0A060YIL0_ONCMY | Uncharacterized protein (Fragment) | 4 | 0.23 | 0.02 | 0.50 | CSF007-82 | 1.9 | **2.3*** | 1.6 |
|  |  |  |  |  |  | 7959-11 | 1.9 | 1.9 | 1.4 |
| A0A060Z4D4_ONCMY | Uncharacterized protein (Fragment) | 4 | 0.46 | 0.03 | 0.73 | CSF007-82 | 1.5 | **2.8*** | -1.1 |
|  |  |  |  |  |  | 7959-11 | 1.6 | 1.9 | 1.1 |
| A0A060WA19_ONCMY | Uncharacterized protein | 2 | 0.03 | 0.13 | 0.32 | CSF007-82 | **2.7*** | 2.3 | 1.9 |
|  |  |  |  |  |  | 7959-11 | 1.8 | 1.8 | 1.1 |
| A0A060Y9N6_ONCMY | Uncharacterized protein (Fragment) | 2 | 0.65 | 0.06 | 0.04 | CSF007-82 | 1.6 | 2.8 | **3.8*** |
|  |  |  |  |  |  | 7959-11 | 1.4 | 2.2 | **2.3*** |
| A0A060YQ22_ONCMY | Uncharacterized protein (Fragment) | 2 | 0.03 | 0.14 | 0.37 | CSF007-82 | 1.6 | 1.8 | 1.5 |
|  |  |  |  |  |  | 7959-11 | **2.5*** | 1.2 | 1.3 |
| A0A060Y7T4_ONCMY | Uncharacterized protein | 2 | 0.45 | 0.03 | 0.35 | CSF007-82 | 1.8 | **2.3*** | 1.3 |
|  |  |  |  |  |  | 7959-11 | 1.8 | 1.6 | 1.1 |
| A0A060YXS6_ONCMY | Uncharacterized protein | 4 | 0.84 | 0.03 | 0.34 | CSF007-82 | 1.2 | **2.2*** | 1.6 |
|  |  |  |  |  |  | 7959-11 | -1.1 | 1.7 | 1.6 |
| A0A060VQZ6_ONCMY | Uncharacterized protein | 6 | 0.04 | 0.08 | 0.12 | CSF007-82 | **-2.4*** | -2.9 | -3.2 |
|  |  |  |  |  |  | 7959-11 | **-2.4*** | -2.5 | -2.4 |
| A0A060W754_ONCMY | Uncharacterized protein | 6 | 0.45 | 0.37 | 0.04 | CSF007-82 | -1.4 | -1.7 | **-2.1*** |
|  |  |  |  |  |  | 7959-11 | -1.1 | -1.1 | -1.7 |
| A0A060W4N0_ONCMY | Uncharacterized protein | 6 | 0.02 | 0.60 | 0.39 | CSF007-82 | **-4.8*** | -3.3 | -1.5 |
|  |  |  |  |  |  | 7959-11 | **-3.8*** | -2.1 | -1.3 |
| A0A060YPN6_ONCMY | Uncharacterized protein | 6 | 0.09 | 0.01 | 0.22 | CSF007-82 | -2.0 | -1.9 | -2.2 |
|  |  |  |  |  |  | 7959-11 | -2.2 | **-2.0*** | -1.5 |
| A0A060WT62_ONCMY | Uncharacterized protein | 4 | 0.61 | 0.01 | 0.65 | CSF007-82 | -1.8 | **-4.9*** | -1.3 |
|  |  |  |  |  |  | 7959-11 | -1.1 | -1.0 | 1.2 |
| A0A060YPS5_ONCMY | Uncharacterized protein | 6 | 0.02 | 0.22 | 0.95 | CSF007-82 | -1.8 | -1.3 | 1.0 |
|  |  |  |  |  |  | 7959-11 | **-2.7*** | -1.2 | -1.0 |
| A0A060X9U4_ONCMY | Uncharacterized protein | 2 | 0.04 | 0.24 | 0.81 | CSF007-82 | -1.8 | -1.5 | -1.2 |
|  |  |  |  |  |  | 7959-11 | **-3.0*** | -1.9 | -1.2 |
| A0A060YYU3_ONCMY | Uncharacterized protein | 2 | 0.02 | 0.72 | 0.37 | CSF007-82 | **-6.0*** | -1.5 | -2.0 |
|  |  |  |  |  |  | 7959-11 | **-3.5*** | 1.0 | -1.1 |
| A0A060WR72_ONCMY | Uncharacterized protein | 2 | 0.05 | 0.09 | 0.35 | CSF007-82 | -1.8 | -1.6 | -1.5 |
|  |  |  |  |  |  | 7959-11 | **-2.1*** | -1.5 | -1.3 |
| A0A060XIH9_ONCMY | Uncharacterized protein | 2 | 0.90 | 0.94 | 0.01 | CSF007-82 | -1.6 | -1.2 | **-3.0*** |
|  |  |  |  |  |  | 7959-11 | -1.6 | -1.1 | **-2.4*** |
| A0A060WP40_ONCMY | Uncharacterized protein | 2 | 0.02 | 0.59 | 0.72 | CSF007-82 | -1.2 | -2.2 | -1.1 |
|  |  |  |  |  |  | 7959-11 | **-4.9*** | -1.1 | -1.4 |
| A0A060XNH5_ONCMY | Uncharacterized protein | 2 | 0.03 | 0.02 | 0.35 | CSF007-82 | **-2.1*** | -1.9 | -1.5 |
|  |  |  |  |  |  | 7959-11 | -1.9 | -2.0 | -1.5 |
