# Supplementary material for: Proteome analysis reveals a role of rainbow trout lymphoid organs during Yersinia ruckeri infection process
Source: Sci Rep. 2018 Sep 18;8:13998. doi: 10.1038/s41598-018-31982-6 (PMC6143608; doi:10.1038/s41598-018-31982-6)
Supplement: Supplementary file 3 — List of protein abbreviation used in the protein-protein interaction network. [file 41598_2018_31982_MOESM3_ESM.doc]

**Proteome analysis reveals a role of rainbow trout lymphoid organs** **during *Yersinia ruckeri* infection process**

Gokhlesh Kumar^1^*, Karin Hummel^2^, Katharina Noebauer^2^, Timothy J Welch^3^, Ebrahim Razzazi-Fazeli^2^ & Mansour El-Matbouli^1^

# ^1^Clinical Division of Fish Medicine, University of Veterinary Medicine, Vienna, Austria

^2^VetCore Facility for Research / Proteomics Unit, University of Veterinary Medicine, Vienna, Austria

^3^National Center for Cool and Cold Water Aquaculture, Kearneysville, USA

*Corresponding Author

**Supplementary Table S3:** List of protein abbreviation used in the protein-protein interaction network. It shows details of protein abbreviation with the percentage of amino acid sequence blasted against zebrafish in Figure 4.

| **STRING protein** | **Abbreviation** | **Identify (%) of amino acid sequence blasted against zebrafish** |
| --- | --- | --- |
| eno1a | Enolase 1a, (alpha) | 94 |
| fbp1a | Fructose-1,6-bisphosphatase 1a | 89 |
| gpx1b | Glutathione peroxidase 1b | 83 |
| rpl36 | Ribosomal protein L36 | 87 |
| ncf2 | Neutrophil cytosolic factor 2 | 65 |
| taldo1 | Transaldolase 1 | 83 |
| lect2l | Leukocyte cell-derived chemotaxin 2 like | 65 |
| pgm3 | Phosphoglucomutase 3 | 81 |
| npc2 | Niemann-Pick disease, type C2 | 71 |
| cd81 | CD81 antigen | 71 |
| snrpf | Small nuclear ribonucleoprotein polypeptide F | 94 |
| rps12 | Ribosomal protein S12 | 99 |
| actb1 | Actin, beta 1 | 99 |
| ddx5 | DEAD (Asp-Glu-Ala-Asp) box polypeptide 5 | 77 |
| ncf4 | Neutrophil cytosolic factor 4 | 74 |
| abracl | ABRA C-terminal like | 81 |
| rps5 | Ribosomal protein S5 | 96 |
| txn | Thioredoxin | 75 |
| tagln | Transgelin | 78 |
| zff9 | Zinc finger protein 9 | 84 |
| tmed7 | Transmembrane emp24 protein transport domain containing 7 | 88 |
| b2m | Beta-2-microglobulin | 61 |
| ctsba | Cathepsin B | 82 |
| pfkfb4 | 6-phosphofructo-2-kinase/fructose-2,6-biphosphatase 4 | 88 |
| lyz | Lysozyme | 43 |
| amt | Aminomethyltransferase | 81 |
| ppp1cab | Protein phosphatase 1, catalytic subunit, alpha isoform b | 94 |
| dynll1 | Dynein, light chain, LC8-type 1 | 100 |
| slc25a5 | Solute carrier family 25 alpha, member 5 | 90 |
| npsn | Nephrosin | 60 |
| hbaa1 | Hemoglobin alpha adult-1 | 69 |
| steap4 | STEAP family member 4 | 68 |
| rac2 | Ras-related C3 botulinum toxin substrate 2 | 98 |
| rps27a | Ribosomal protein S27a | 99 |
| pkma | Pyruvate kinase, muscle | 84 |
| cars | cysteinyl-tRNA synthetase | 82 |
| g6pd | Glucose-6-phosphate dehydrogenase | 90 |
| stoml3b | Stomatin (EPB72)-like 3b | 81 |
