# Supplementary material for: Proteome analysis reveals a role of rainbow trout lymphoid organs during Yersinia ruckeri infection process
Source: Sci Rep. 2018 Sep 18;8:13998. doi: 10.1038/s41598-018-31982-6 (PMC6143608; doi:10.1038/s41598-018-31982-6)
Supplement: Supplementary file 4 — List of quantitative real-time PCR primers. [file 41598_2018_31982_MOESM4_ESM.doc]

**Proteome analysis reveals a role of rainbow trout lymphoid organs** **during *Yersinia ruckeri* infection process**

Gokhlesh Kumar^1^*, Karin Hummel^2^, Katharina Noebauer^2^, Timothy J Welch^3^, Ebrahim Razzazi-Fazeli^2^ & Mansour El-Matbouli^1^

# ^1^Clinical Division of Fish Medicine, University of Veterinary Medicine, Vienna, Austria

^2^VetCore Facility for Research / Proteomics Unit, University of Veterinary Medicine, Vienna, Austria

^3^National Center for Cool and Cold Water Aquaculture, Kearneysville, USA

*Corresponding Author

**Supplementary Table S4:** List of quantitative real-time PCR primers. PCR primers specific for the selected genes were designed using NCBI Primer BLAST software and used in this study to confirm expression data.

| Primer code | Sequence (5`-3`) | Amplicon size (bp) | Annealing temperature (°C) | NCBI accession no. | Reference |
| --- | --- | --- | --- | --- | --- |
| Lysozyme II F | GCTGTTGTTGTTCTCCTGCT | 129 | 57 | NM_001124716.1 | In this study |
| Lysozyme II R | GCAAACCCAGTTGGGCAG |
| Chemotaxin F | GGCTTTACCATGAAGACTGCTG | 172 | 57 | NM_001124309.1 | In this study |
| Chemotaxin R | CACGGCCTCCTCTGCTT |
| S100 F | GCCTTCTCTCCTGAAGGCTTCTAA | 162 | 58 | XM_021578278.1 | In this study |
| S100 R | GGAGCTCATTTCTTGGGGCA |
| Thioredoxin F | AGACAAGCTGGTGGTAGTGGAC | 121 | 57 | XM_021577868.1 | In this study |
| Thioredoxin R | CACCTTGAGGAAAACCACGTTAC |
| p40phox F | TAAAGGGGCGGGGTCTTACA | 161 | 57 | NM_001124505.1 | In this study |
| p40phox R | CGATGTTGCCATCCACCTTG |
| MHC I alpha F | CAGGTGTGCACGTTTTCCAG | 163 | 57 | JZ713068 | Kumar *et al.*^54^ |
| MHC I alpha R | TTGGTGATGACTGCCTGTGG |
| Beta-actin F | ATGGAAGGTGAAATCGCC | 260 | 53 | AF157514 | Rucker & El-Matbouli* |
| Beta-actin R | TGCCAGATCTTCTCCATG |
| EF-1α F | AGACAGCAAAAACGACCCCC | 167 | 57 | HF563594 | Kumar *et al.*^54^ |
| EF-1α R | AACGACGGTCGATCTTCTCC |

*Rucker, U. & El-Matbouli, M. Sequence analysis of OmNramp α and quantitative expression of Nramp homologues in different trout

strains after infection with *Myxobolus cerebralis*. *Dis. Aquat. Organ.* **76**, 223–230 (2007).
